# Synthesis and Functionalization of Graphene Materials for Biomedical Applications: Recent Advances, Challenges, and Perspectives

**DOI:** 10.1002/advs.202205292

**Published:** 2023-01-19

**Authors:** Yuqin Xiao, Yoong Xin Pang, Yuxin Yan, Ping Qian, Haitao Zhao, Sivakumar Manickam, Tao Wu, Cheng Heng Pang

**Affiliations:** ^1^ Department of Chemical and Environmental Engineering University of Nottingham Ningbo China Ningbo 315100 P. R. China; ^2^ New Materials Institute University of Nottingham Ningbo 315100 P. R. China; ^3^ Materials Interfaces Center Shenzhen Institute of Advanced Technology Chinese Academy of Sciences Shenzhen Guangdong 518055 P. R. China; ^4^ College of Energy Engineering Zhejiang University Hangzhou Zhejiang 310027 P. R. China; ^5^ Beijing Advanced Innovation Center for Materials Genome Engineering Beijing 100083 P. R. China; ^6^ School of Mathematics and Physics University of Science and Technology Beijing Beijing 100083 P. R. China; ^7^ Petroleum and Chemical Engineering Faculty of Engineering Universiti Teknologi Brunei Bandar Seri Begawan BE1410 Brunei Darussalam; ^8^ Key Laboratory for Carbonaceous Wastes Processing and Process Intensification Research of Zhejiang Province University of Nottingham Ningbo China Ningbo 315100 P. R. China; ^9^ Municipal Key Laboratory of Clean Energy Conversion Technologies University of Nottingham Ningbo China Ningbo 315100 P. R. China

**Keywords:** biocompatibility, characterization techniques, functionalization process, graphene‐based materials, synthesis methods

## Abstract

Since its discovery in 2004, graphene is increasingly applied in various fields owing to its unique properties. Graphene application in the biomedical domain is promising and intriguing as an emerging 2D material with a high surface area, good mechanical properties, and unrivalled electronic and physical properties. This review summarizes six typical synthesis methods to fabricate pristine graphene (p‐G), graphene oxide (GO), and reduced graphene oxide (rGO), followed by characterization techniques to examine the obtained graphene materials. As bare graphene is generally undesirable in vivo and in vitro, functionalization methods to reduce toxicity, increase biocompatibility, and provide more functionalities are demonstrated. Subsequently, in vivo and in vitro behaviors of various bare and functionalized graphene materials are discussed to evaluate the functionalization effects. Reasonable control of dose (<20 mg kg^−1^), sizes (50–1000 nm), and functionalization methods for in vivo application are advantageous. Then, the key biomedical applications based on graphene materials are discussed, coupled with the current challenges and outlooks of this growing field. In a broader sense, this review provides a comprehensive discussion on the synthesis, characterization, functionalization, evaluation, and application of p‐G, GO, and rGO in the biomedical field, highlighting their recent advances and potential.

## Graphical abstract

This article is a comprehensive review on the synthesis, characterization, functionalization, toxicity/biocompatibility, and potential of graphene materials in biomedical application. It critically discusses the typical synthesis methods to fabricate graphene materials suited for biomedical applications, followed by a range of structural and functional characterization techniques to characterize the obtained graphene materials. The challenges, potential solutions, and perspectives are also discussed.

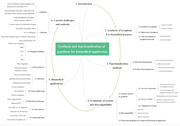

## 1 Introduction

Graphene is a single or few atoms thick sheet of sp^2^‐bonded carbon atoms in a closely packed honeycomb 2D lattice. Naked graphene materials, including pristine graphene (p‐G), graphene oxide (GO), and reduced graphene oxide (rGO), were reported to obtain unique properties such as large surface area,^[^ ^1^ ^]^ high absorption of laser,^[^ ^2^ ^]^ high elasticity,^[^ ^3^ ^]^ good charge‐transfer ability,^[^ ^4^ ^]^ and ferromagnetic properties.^[^ ^5^ ^]^ Thus, graphene materials have gained wide popularity in biomedical applications, including medical imaging,^[^ ^6^ ^]^ drug/gene delivery,^[^ ^7^ ^]^ photothermal therapy,^[^ ^8^ ^]^ tissue engineering,^[^ ^9^ ^]^ biosensing,^[^ ^10^ ^]^ and immunotherapy.^[^ ^11^ ^]^ However, the toxicity of naked graphene materials and their poor biocompatibility impede related research.^[^ ^12^ ^]^ To solve this problem, both covalent^[^ ^13^ ^]^ and noncovalent^[^ ^14^ ^]^ functionalization methods are utilized to not only reduce toxicity and improve biocompatibility by using polymers such as poly(ethylene glycol) (PEG),^[^ ^15^ ^]^ poly(vinyl alcohol) (PVA)^[^ ^16^ ^]^ but also to functionalize graphene‐based materials with various biomolecules.^[^ ^17^ ^]^ Though the application of graphene materials in the biomedical sector has been developing rapidly in recent years, several challenges remain to be addressed before their commercialization for biomedical use.

Existing published reviews on graphene and its derivatives generally include individual synthesis strategies,^[^ ^18^ ^]^ functionalization methods,^[^ ^19^ ^]^ toxicity,^[^ ^20^ ^]^ and their applications in the biomedical sector, particularly in biomedical imaging,^[^ ^21^ ^]^ and drug delivery.^[^ ^22^ ^]^ This review expands on the previously published work and provides a comprehensive overview of the life cycle of graphene materials, comprising the synthesis processes, characterization and analysis, functionalization methods, toxicity and biocompatibility evaluations, as well as the applications of p‐G, GO, and rGO in various biomedical areas.

**Figure 1:**
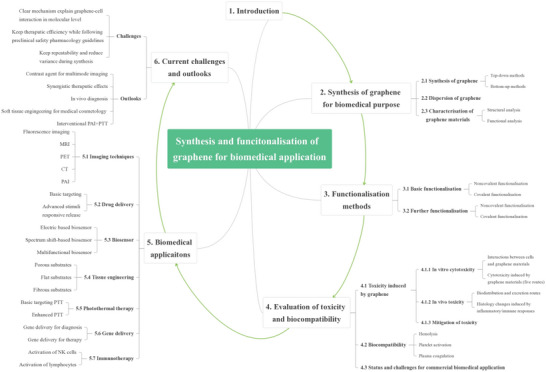
Illustration of the structure and content of this review.

The whole structure and content of this review is demonstrated in **Figure** **1**. This review introduces six typical methods to synthesize p‐G, GO, and rGO for biomedical applications, and suitable improvement strategies are proposed at the end of each section. Then, the characterization techniques generally utilized to observe the synthesized graphene's structure, functional groups, and properties are demonstrated. Then, basic and further functionalization methods involving covalent and noncovalent routes to improve the behavior of graphene materials during biomedical applications are presented. Also, the toxicity of in vitro and in vivo and the biocompatibility of graphene materials are elucidated. The following summarizes seven areas of biomedical applications, including imaging techniques, drug delivery, biosensing, tissue engineering, photothermal therapy, gene delivery, and immunotherapy based on graphene materials. Furthermore, comparative and balanced views of current challenges and further prospective applications of graphene materials have been demonstrated.

## 2 Synthesis of Graphene for Biomedical Applications

Various synthesis methods have been employed to produce graphene materials with different properties. Understanding the synthesis methods, postprocessing (dispersion), and characterization techniques would benefit medical and biological scientists when choosing suitable graphene materials from an initial stage during the exploration of biomedical applications. This section provides an overview of the preparation of graphene for biomedical applications from the aspects of synthesis, dispersion, and characterization. Typical approaches to fabricating graphene for biomedical applications are involved in the first part, including top‐down and bottom‐up methods. After synthesizing graphene, the graphene materials are generally dispersed or separated in a polar solvent to obtain a uniform distribution, which benefits further functionalization and provides a uniform dose during applications. Characterization techniques are categorized into structural and functional analyses and can be applied to observe and evaluate the prepared graphene‐based materials.

### 2.1 Synthesis of Graphene

Synthesis methods have been comprehensively reviewed and summarized from various dimensions,^[^ ^18^, ^23^ ^]^ especially scalable production, classification, and application of graphene have been thoroughly reviewed.^[^ ^23c^ ^]^ In targeting biomedical applications, large‐scale synthesis of graphene with high purity, easy dispersion, and functionalization is preferred. Therefore, synthesis methods focusing on fabricating graphene with more than 200 mg or films larger than 200 cm^2[^ ^23c^ ^]^ and generating little harmful impurities are discussed in this section. This section provides an overview of the six approaches to fabricating graphene for biomedical applications, where top‐down methods include mechanical exfoliation, chemical exfoliation, reduction of graphene oxide, and bottom‐up methods involving chemical vapor deposition, arc discharge, and flash heating are introduced.

#### 2.1.1 Top‐Down Methods

##### Mechanical Exfoliation

Mechanical exfoliation separates stacked layers by overcoming the van der Waals (vdW) forces through mechanical force.^[^ ^5^ ^]^ The improving trend in the last decade of mechanical exfoliation could be summarized as from small‐scale repeated scotch peeling^[^ ^1^, ^18^ ^]^ to large‐scale microwave,^[^ ^24^ ^]^ ultrasound,^[^ ^25^ ^]^ or shear mixing^[^ ^26^ ^]^ assisted mechanical exfoliation. Recent advancements have applied these mechanically assisted exfoliation methods to synthesize graphene as raw materials in large‐scale for biomedical applications. For instance, Pang's group described an extrinsic corrugation‐assisted mechanical exfoliation (ECAME) synthesizing monolayer graphene on SiO_2_ substrate.^[^ ^24^ ^]^ With thermal treatment, the graphene–SiO_2_ adhesion energy overcomes the graphene layers; under such an energy‐dependent manner, monolayer graphene could be fractured after removing the top graphite flake with scotch tape. The ECAME method allows large‐scale single‐layer graphene (SLG) production that exhibits great potential as a key material for biosensors after further functionalization. Similarly, Pirzado et al. investigated on mechanical exfoliation of pure graphite discs on a rough glass surface to obtain FLG.^[^ ^27^ ^]^ It is a high‐yield method with a simplified set‐up and fewer purification steps (the total process time is ≈8 h). Paton et al. first proposed a high shear mixing method for large‐scale generation of high‐quality graphene without adding surfactant or dispersant for intercalation, where the shear rate exceeded 10^4^ s^−1^, and 100 g h^−1^ of graphene was generated using an equipment volume of 10 m^3^.^[^ ^26^ ^]^ Under centrifugal extrusion, liquid layer friction, and hydraulic‐induced forces, graphene was exfoliated from graphite, where graphene generation was many times more efficient than sonication, and it can be scaled up to an industrial level. Despite achieving relatively high productivity and quality, the exfoliated graphene was almost pristine (p‐G).^[^ ^28^ ^]^ Hydrophobic p‐G showed low biocompatibility and is relatively toxic in vivo, as discussed in Section 4; thus, the production still requires further control of functional groups^[^ ^29^ ^]^ and lateral size^[^ ^30^ ^]^ before its utilization in the biomedical field.

##### Chemical Exfoliation

Chemical exfoliation is a two‐step process where intercalating agents such as potassium permanganate (KMnO_4_) oxidant,^[^ ^31^ ^]^ alkali metal ions,^[^ ^32^ ^]^ dodecyl amine organic molecules,^[^ ^12a^ ^]^ and supercritical CO_2_^[^ ^33^ ^]^ are first used to increase the interlayer spacing of graphite. The graphene‐intercalated compounds are subsequently exfoliated into FLG sheets by heating,^[^ ^1^ ^]^ sonication^[^ ^5^ ^]^ or shear mixing^[^ ^34^ ^]^ in the second step. As oxidation via the introduction of oxygen atoms is the most common method to increase the interlayer spacing of graphite,^[^ ^31^, ^35^ ^]^ chemical exfoliation generally produces GO and rGO. Betancur et al. utilized ammonium hydroxide as an intercalating agent and synthesized rGO directly from mineral graphite via chemical exfoliation.^[^ ^31^ ^]^ Gebreegziabher's group proposed a one‐step chemical route for synthesizing water‐soluble rGO at the thickness of 0.36 nm using sulfuric acid (H_2_SO_4_)/potassium permanganate (KMnO_4_) assisted by ultrasonication. The temperature during exfoliation also influences GO reduction, where GO was produced at 60 °C.

In comparison, rGO was synthesized under the same conditions except at 120 °C.^[^ ^35^ ^]^ Alkali metal ions such as potassium, sodium, and lithium can be applied as intercalating agents. Considering the bonding in the vertical direction is relatively weak, the interlayer spacing of 3.35 Å can support guest ion intercalation based on host–guest chemistry.^[^ ^32^ ^]^ Organic molecules like tetraoctylammonium bromide (TOAB),^[^ ^36^ ^]^ hydrophobin (HFBI),^[^ ^37^ ^]^ and dodecyl amine^[^ ^12^, ^30^ ^]^ were reported to intercalate graphite via different pathways which related to the properties of molecules. Ionic organic molecules such as TOAB intercalate through electrochemical interactions under different reduction potentials,^[^ ^36^ ^]^ and hydrophobic HFBI interacts strongly with hydrophobic surfaces of graphite.^[^ ^37^ ^]^

By contrast, polar organic molecules like dodecylamine intercalate through the host–guest chemistry, similar to alkali metal ions.^[^ ^12a^ ^]^ Chemical exfoliation facilitates large‐scale graphene production, but dispersing graphene materials during intercalation may involve toxic solvents like N‐methylpyrrolidone (NMP), which are harmful to the environment and the human body. Additionally, the long processing time (on average 26 h)^[^ ^31^, ^32^ ^]^ of chemical exfoliation is also a challenge to address for the efficient production of graphene materials. Wang et al. generated large‐scale graphene suitable for biomedical application, where they applied ultrasound (250 W) coupled with a shear mixer (4000 rpm) in supercritical CO_2_ with a reaction time of 180 min.^[^ ^33^ ^]^ Graphene was generated with a high yield (82.6%), and ≈60% of the graphene sheets were obtained in less than three layers. In this process, supercritical CO_2_, due to its low critical point and small molecular size, acts as an excellent interactive medium that assists graphene's chemical exfoliation with high efficiency and safety without any residue. Moreover, ultrasonication activated the edges of the graphite and enhanced the gap between layers while associated with the exfoliation of graphene from the gap based on shear mixing. Therefore, it is necessary to reduce the toxicity of the as‐produced graphene to the living body based on suitable intercalation molecules. Also, it is important to increase the efficiency based on assisted exfoliation processes such as ultrasonication, microwave heating or shear mixing to synthesize graphene materials targeted for biomedical applications.

##### GO Reduction

GO reduction is applied to generate graphene materials, rGO, that possess medium oxygen‐containing functional groups between p‐G and GO, as p‐G and GO damage cells physically.^[^ ^38^ ^]^ By contrast, rGO induces less membrane damage attributed to a low oxidation state, and few reactive surface groups are applied as raw graphene materials for biomedical applications.^[^ ^39^ ^]^ This method involves two steps: synthesizing GO from graphite, reducing GO, and producing rGO.^[^ ^40^ ^]^ Brodie's oxidation method, Staudenmaier method, Hofmann method,^[^ ^41^ ^]^ and Hummer's method^[^ ^42^ ^]^ were reported as oxidation methods, which generate toxic gases due to the application of strong oxidants, including fuming HNO_3_, concentrated H_2_SO_4_, and the process is also relatively time‐consuming. Modified Hummer's and rapid heating methods are more likely to be applied, considering the efficiency and hazards. Modified Hummer's method changes the ratio between NaNO_3_ and KMnO_4_, and the mixture is modified (H_2_SO_4_ to H_2_SO_4_/H_3_PO_4_ or H_2_CrO_4_/H_2_SO_4_) to conduct the reaction.^[^ ^23^, ^43^ ^]^ Recently, several studies have applied rapid heating methods,^[^ ^44^ ^]^ where KMnO_4_ is excluded. The pure nitronium ion oxidation was combined with microwave heating to convert *π*‐conjugated aromatic structures and associated unique properties. Chemical, electric, and thermal reduction methods commonly reduce GO to obtain rGO. Applying chemical reductants that do not react with water is traditional.^[^ ^1^, ^45^ ^]^ For example, hydrazine monohydrate,^[^ ^14^ ^]^ hydrazine hydrate,^[^ ^46^ ^]^ a large excess of NaBH_4_,^[^ ^47^ ^]^ phenylhydrazine,^[^ ^40^ ^]^ hydroxylamine,^[^ ^41^ ^]^ glucose,^[^ ^23b^ ^]^ ascorbic acid,^[^ ^45^, ^48^ ^]^ proteins,^[^ ^49^ ^]^ and novel green‐reductant like delphinium root extract^[^ ^50^ ^]^ were utilized as reductants. Electrochemical reduction is another reducing method, where rGO was demonstrated to precipitate on the graphite electrode with a voltage change from −0.8 to −1.5 V.^[^ ^51^ ^]^ This method is relatively clean and is based on the reaction of GO + H^+^ + e^−^ = rGO + H_2_O.^[^ ^52^ ^]^ The third pathway is based on thermal reduction, either dispersing GO in an organic solvent like dimethylformamide,^[^ ^53^ ^]^ dimethyl sulfoxide,^[^ ^40^ ^]^ NMP,^[^ ^54^ ^]^ and then heating to the boiling point under reflux conditions or relatively clean and rapid photothermal and photochemical treatment that applies UV, Xenon flash, and KrF excimer laser.^[^ ^55^ ^]^ In conclusion, researchers tend to study methods that produce less residual gas and require less time on the first step of GO synthesis. Moreover, in the second reduction step, rGO produced differently shows various qualities. The products must be dispersible, biocompatible, and less toxic for biomedical applications. Therefore, toxic chemical reductants should not be involved in this process, and electron/laser‐related methods seem to be a good enhancer for saving time.

#### 2.1.2 Bottom‐Up Methods

##### Chemical Vapor Deposition

Chemical vapor deposition (CVD) is widely reported in the synthesis of graphene materials, where the basic principle is to deposit graphene materials on different substrates^[^ ^23^, ^56^ ^]^ by utilizing hydrocarbon or other carbon‐bearing precursors^[^ ^17^, ^57^ ^]^ in the presence of a catalyst under various atmospheres.^[^ ^9^, ^58^ ^]^ There are plenty of classifications of CVD based on the differences in the substrate, carbon source, pressure setting, and enhancing methods. Traditional CVD based on metal substrates has drawbacks as there would be loss of product, corrugation, contamination, and breakage of graphene samples during product collection from metal substrates. Graphene generated from CVD for biomedical applications is supposed to obtain a larger size and be free of transfer from substrates if applied as a biosensor; the adjustable layers and synthesis with lower temperature and less time would also be beneficial. Several modified CVD methods were proposed targeting these points, and it is noticed that existing CVD technology could achieve large‐scale production^[^ ^18^, ^56^ ^]^ of high‐quality graphene sheets with a controllable number of layers.^[^ ^59^ ^]^ Chen's group demonstrated a near‐equilibrium CVD method that produces large‐scale graphene sheets on various dielectric substrates.^[^ ^56a^ ^]^ Compared with metal substrates, due to the relatively higher energy barrier on dielectric substrates, the diffusion of carbon precursors on the dielectric surface does not affect the evolution of the graphene shape. Thus, the graphene is tested to be highly crystalline with clean, wrinkle‐free, breakage‐free morphology and obtain high carrier mobility (>5000 cm^2^ V ^−1^ s ^−1^). Results have also shown that with increasing growth time from 9 to 72 h, the lateral size of graphene increases up to 11 µm, indicating a large‐scale graphene production through this method. Tu's group proposed a CVD method based on Cu substrate to control the graphene layer synthesized by adjusting parameters including the reaction pressure, the flow ratio of CH_4_ and H_2_, the temperature, and the reaction time.^[^ ^59^ ^]^ In their results, graphene with 1–2 layers was produced with low pressure, multilayer (3–7) graphene was synthesized with atmosphere pressure, and the other three parameters were modified based on the applied pressures. Molecular dynamics simulation can probably predict suitable reaction parameters for the specific graphene layer instead of trial and error. Traditional CVD generally needs a high temperature (900–1000 °C).^[^ ^56b^ ^]^ Several recent studies used plasma‐enhancing methods to produce graphene at a lower reaction temperature and reduce production costs.^[^ ^18^, ^23^ ^]^ Compared with traditional CVD, this method provided a deposition time of less than 5 min and a lower growth temperature of 650 °C.^[^ ^18^ ^]^

##### Arc Discharge Method

The arc discharge method generally applies pure graphite rods as anode and cathode, immersed in an inert gas like He.^[^ ^60^ ^]^ With a direct‐current arc voltage applied across two graphite electrodes, the graphite in the anode evaporates, and graphene deposits on the cathode.^[^ ^61^ ^]^ The arc evaporation of graphite in the presence of hydrogen could produce graphene sheets with two or three layers and a lateral size of 100–200 nm.^[^ ^5^ ^]^ Studies have also explored other starting materials to synthesize B^[^ ^1^ ^]^ or N^[^ ^62^ ^]^ doped graphene. In the early stage, small‐size and low‐quality graphene flakes were produced through the arc‐discharge method under relatively high hydrogen pressure and without any catalyst.^[^ ^5^, ^61^ ^]^ Therefore, modified methods changed H_2_ to CO_2_,^[^ ^63^ ^]^ NH_3_,^[^ ^62^ ^]^ or other mixtures^[^ ^29^, ^38^, ^60^ ^]^ to synthesize graphene or nitrogen‐doped graphene sheets more suitable for biomedical applications. The catalyst was found to affect the generated graphene layer, where Huang's group performed four experiments with different catalysts and carbon sources to investigate graphene productivity.^[^ ^63^ ^]^ It was reported that flake graphite produces gram‐scale and high‐quality few‐layer graphene sheets when catalyzed by zinc‐based catalysts (ZnO or ZnS). However, the graphene layers decreased when the experiment was repeated with Cu powder as a catalyst. As SiC was utilized as a carbon source, the synthesized graphene was mainly of one to three layers. However, the drawback of the arc discharge method is the nonuniform distribution of graphene; Borand et al. noticed that the deposits collected from the cathode and inner wall differed. They studied the formation mechanism and proposed that the interlayer spacing, crystallite size, number of average layers, and defect density varied with the position of the stainless‐steel reactor.^[^ ^60^ ^]^ Also, they showed that the interlayer spacing, crystallite size, and the average number of graphene layers collected from the anode region were larger than the front region. Graphene from the left‐side region of the reactor indicated the smallest value, while the defect density values exhibited an opposite tendency among the three regions. The formation may be influenced by the charge, and inert gas distribution in the reactor, perhaps further theoretical study of the charge distribution and heat and mass transformation during graphene formation is needed to solve the problem of nonuniform distribution of graphene during arc discharge synthesis; it would also be a solution that applies a sorting process like centrifugation based on the mass of graphene sheets to obtain uniform sized graphene.

##### Flash Heating Method

The flash heating method includes flash Joule heating (FJH) and laser radiation, where a rapid temperature increases, and cooling is involved. FJH, with its great potential, results in the high‐quality, large‐scale generation of graphene without applying solvents or energy‐consuming exfoliation. It was first reported by Luong et al., where the high voltage discharge from the capacitor bank allows the carbon source to reach temperatures above 3000 K in less than 100 ms, effectively converting amorphous carbon into turbostratic graphene.^[^ ^64^ ^]^ Stanford et al. further explored the mechanisms and found that when high voltage is applied, the current travels along the path with the least resistance, thus providing more energy for the conducting area, thereby significantly annealing and graphitizing these areas. With the gradually increasing conductivity, the formation of conductive channels increases and a turbostratic graphene sheet is formed.^[^ ^65^ ^]^ The factors affecting graphene generation in FJH require further study before achieving high‐efficiency synthesis of specific graphene. Apart from the influence of carbon sources,^[^ ^64^ ^]^ heating duration is also important, according to Chen et al.^[^ ^66^ ^]^ Energy is increased with an increase in heating duration, and a phase change occurs from fluorinated amorphous carbon (≈10 ms) to fluorinated nanodiamonds (≈35 ms), fluorinated turbostratic graphene (≈100 ms), and fluorinated concentric carbon (≈500 ms). However, there is still no specific corresponding energy requirement for the formation of each structure; perhaps a molecular dynamics study based on the reactive force field could be beneficial to such studies. Another interesting study by Beckham et al. autonomously improved the crystallinity of flash graphene over many trials with machine learning based on the XGBoost regression model, where five features were chosen, and Raman spectral mapping was used as the training dataset.^[^ ^67^ ^]^ It is probably properties except crystallinity could be improved based on expanded ML or deep learning methods without manually choosing features if with a large enough dataset and well‐established neural networks.

In terms of laser‐induced graphene, sharply increased temperature from the laser breaks the chemical bonds and rearranges the carbon atom. Graphene materials are formed during the cooling process, where the reaction atmosphere, laser wavelength, light source, and starting material composition influence the graphene properties.^[^ ^68^ ^]^ Substrates of quartz^[^ ^69^ ^]^ and wood^[^ ^70^ ^]^ were applied in an early study; for instance, Ye et al. transformed wood into hierarchical porous laser‐induced graphene, where CO_2_ as a working substance induced laser with a wavelength of 10.6 µm.^[^ ^70^ ^]^ The laser obtained a maximum power of 75 W, and the graphene layer decreased with increased power. The laser‐induced graphene was found to be GO based on X‐ray photoelectron spectroscopy (XPS) characterization, attributed to the abundant oxygen‐containing functional groups in cellulose, hemicellulose, and lignin. Though the method is facile, and the size or layers of graphene are controllable, the laser‐induced graphene generated from wood etching required removal from the wood surface for further application. Thus, modification of the methods seems necessary for biomedical applications. Luong et al. proposed a one‐step infiltration process to solve this problem.^[^ ^34^ ^]^ The laser‐induced graphene was initially generated from polyimide film. In the infiltration process, hydrophobic materials such as polyethylene and wax infiltrated the laser‐induced graphene. After the polyimide film was removed, the laser‐induced graphene composites were synthesized. The application of such composite for thermal therapy was also tested in their study. Apart from the infiltration process for graphene transfer, it is probably uniformly distributed biomass powders with hundreds of micrometers of size radiated by high power laser in an inert gas atmosphere for a short duration that may also be a solution to generate transfer‐free graphene powder directly.

### 2.2 Dispersion of Graphene

Dispersion of graphene in solvents must be achieved at a certain concentration and maintain stability for a reasonable period targeting the final applications.^[^ ^71^ ^]^ In terms of the dispersion mechanisms, graphene sheets aggregate based on vdW or *π*–*π* stacking; when there are interactions between solvents and graphene, the overlap of electron clouds could bring steric effects, causing tensions that overcome the interaction between sheets and pass the energy barrier from surface energy. Furthermore, graphene sheets are finely dispersed in the solvents. However, the performance of overlapped electron clouds induces steric effects, and the steric repulsion further induces tensions related to the property of solvents and graphene, specifically, the functional groups on the graphene surface and solvent particles. Dispersibility was reported to follow the trend: GO > rGO > p‐G due to the difference in the amount of oxygen‐containing functional groups on graphene; for instance, electrostatic repulsion of carboxylate groups brings the best dispersibility for GO.^[^ ^71a^ ^]^ Additionally, the performance of solvents varied with the defects on the graphene surface, where water could be applied as solvents for rGO or GO,^[^ ^44^, ^57^ ^]^ while p‐G was hard to be dispersed in water.^[^ ^71a^ ^]^ However, specific solvents are favored under certain applications, and the dispersion efficiency is anticipated to increase. Therefore, dispersion assisting methods include microwave heating,^[^ ^44^ ^]^ sonication,^[^ ^5^ ^]^ shear mixing,^[^ ^33^ ^]^ and dispersants or surfactants.^[^ ^43^ ^]^ The application of surfactants is introduced in Section 3 as a functionalization method; other dispersion methods include microwave heating, sonication, and shear mixing that target the preparation of graphene solvents for biomedical applications are introduced in this part.

Low oxygen‐containing GO, and graphene quantum dots were dispersed based on microwave heating. For the mechanism of microwave heating enhanced dispersion, the microwave energy transforms into thermal energy due to the *π* electron mobility in graphite or graphene. Then, the oxygen‐containing functional groups or solvent particles inside the layer soon decompose into volatiles, increasing the interlayer pressure and providing energy to disperse the graphene sheets. The power and irradiation duration influence the shape and concentration of dispersed graphene. Patel et al. synthesized microwave‐enabled low oxygen graphene for photoacoustic imaging, where microwave irradiation was applied for 30 s at 300 W. The graphene concentration in water was 0.4 mg mL^−1^ and kept undisturbed for five days.^[^ ^44^ ^]^ Tak et al. applied higher energy (900 W) for a longer time (5–10 min), where graphene quantum dots instead of graphene sheets were obtained, which may be because of higher energy decomposing the sheets.^[^ ^72^ ^]^ They provided the concentration in mm (16 mm mL^−1^); if converted into mg, it is ≈11 mg mL^−1^. Gürünlü et al. applied a one‐pot synthesis of GO sheets based on 100 mg mL^−1^ graphite solution assisted with microwave heating (180 °C, 150 W for 30 min),^[^ ^73^ ^]^ and an optimum range for surface tension was determined at 45 mN m^−1^ according to their results. The irradiation duration and graphene concentration were positively correlated but require further confirmation. It was noticed that microwave heating was applied with a lower frequency than dispersion using mechanical methods such as shear mixing or ultrasonication, which may be attributed to heating that would induce the reduction of graphene sheets. Long‐term irradiation may lead to decreasing the sheet size.

p‐G, GO, and rGO were dispersed with the assistance of sonication based on the mechanism of the creation of shear stresses and cavitation in the solvent.^[^ ^71a^ ^]^ However, the drawback is the requirement for longer sonication time. Several studies reported dispersion of graphene materials using PEG solution, where 1 mg mL^−1^ aqueous solution of PEG‐6000 and an aqueous suspension of rGO were sonicated for 30 min at room temperature.^[^ ^74^ ^]^ In another study, 2 h sonication was applied for GO in an ice bath, and a concentration of 0.5 mg mL^−1^ was obtained.^[^ ^43^ ^]^ GO dispersion reported by other researchers presented a similar experimental setting of sonication duration.^[^ ^75^ ^]^ A study of synthesis p‐G‐poly(vinyl alcohol) microbubble applied 90 min ultrasonication (320 W) in an aqueous solution of glycolic acid ethoxylate‐4‐nonyphenyl ether,^[^ ^25^ ^]^ and the composite was stable at a concentration of 0.25 mg mL^−1^. It was interesting to find that though GO obtained better dispersity, longer sonication duration was applied for GO compared with rGO and p‐G; perhaps a study of optimal sonication time for graphene with different functional groups would be beneficial to increase the dispersion efficiency. Another drawback of sonication is the nonuniform distribution of graphene size after sonicating for a longer time,^[^ ^7^ ^]^ where a postprocess of centrifugation may be required to produce uniform graphene sheet size. Dispersion of graphene materials in polymer based on only sonication may not be effective when pretreatment of graphene materials is required. rGO could be a representative, and it may be unstable and reaggregate in a polymer matrix even with sonication during dispersion;^[^ ^76^ ^]^ thus, pretreatment of rGO is needed when targeting dispersed graphene in such a polymer matrix. For instance, rGO microsphere aerogels were dispersed better than rGO in polydimethylsiloxane (PDMS) composite with the assistance of ultrasonication,^[^ ^77^ ^]^ attributed to the transformation of rGO from a flat to a spherical shape. The composite was found to have great potential in wound dressing.

Shear mixing was proposed to be a more scalable route to disperse graphene compared with sonication,^[^ ^71a^ ^]^ as both the concentration (8.6 mg mL^−1^) and graphene lateral size (30% yields of few‐layer graphene with good lateral size) were reported to be larger than obtained from sonication.^[^ ^78^ ^]^ In terms of the dispersion mechanism, the Taylor–Couette flow generated from the shear flow between a rotating inner cylinder and a concentric, fixed outer cylinder induces shear stress and separates graphene sheets.^[^ ^23c^ ^]^ It is probably the Taylor–Couette flow in shear mixing that mainly applies shear stress for dispersion, which avoids inducing cavitation and is beneficial to bring less defect on graphene and retain the original lateral size. However, recent studies only reported dispersion of p‐G;^[^ ^26^, ^33^ ^]^ the optimum shear rates and shear duration for dispersion of GO and rGO with different solvents may need further study.

### 2.3 Characterization of Graphene Materials

Various characterization techniques can be applied to observe and evaluate the synthesized graphene‐based materials. The characterization techniques are categorized into structural and functional analyses. Morphology (thickness, lateral size, and defects), dispersion behaviors, and functional groups can be characterized by structural analysis techniques. Functional analyses can be applied to analyze intrinsic properties (mechanical, electronic, laser absorption, ferromagnetic properties, and thermal stability) and extrinsic properties of dynamic behaviors in the physiological environment. Section 2.3 briefly introduces the basic principle of analysis techniques, coupled with a detailed discussion on the required properties and characteristics of graphene materials for biomedical applications.

#### 2.3.1 Structural Analysis

##### Morphology

The layer and size of graphene determine the behavior of graphene materials for drug or gene delivery in biomedical applications. Under the same concentration, fewer layers or larger sizes bring a larger surface area for carrying drugs or genes. Additionally, the toxicity and biocompatibility of graphene materials were reported to be influenced by the size;^[^ ^46^, ^79^ ^]^ thus, it is important to evaluate the layer and size of graphene materials. Raman spectroscopy, scanning electron microscopy (SEM), transmission electron microscopy (TEM), scanning tunneling microscopy (STM) and atomic force microscopy (AFM) can be applied for the quantification of layers. Similarly, size can be evaluated based on SEM, TEM, and AFM. Defects in graphene include intrinsic defects (Stone–Wales defects, single vacancy defects, multiple vacancy defects, line defects, and carbon adatoms)^[^ ^80^ ^]^ and extrinsic defects (mainly oxygen‐containing functional groups on the surface of graphene).^[^ ^81^ ^]^ Stone–Wales defects benefit from increasing the electronic conductivity of graphene when working as biosensors,^[^ ^82^ ^]^ while extrinsic defects could work as sites for functionalization;^[^ ^43^ ^]^ thus, defects are not undesirable in graphene for specific biomedical applications. STM, TEM, AFM, and Raman microscopy can be used to detect defects.

**Figure 2:**
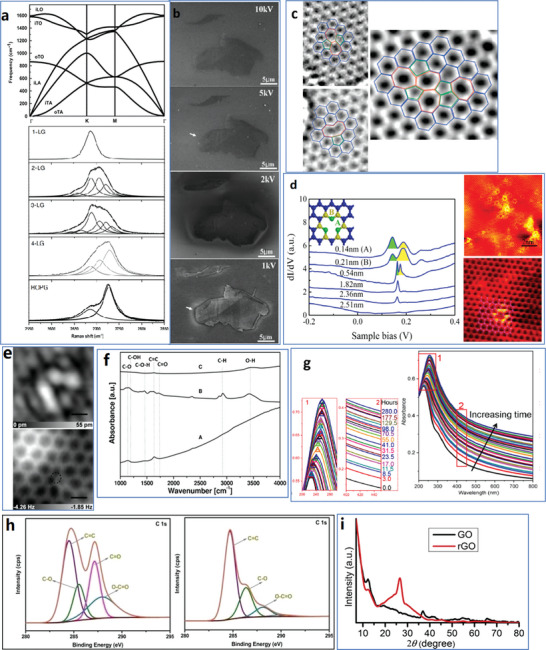
Typical examples of structural detection techniques to analyze graphene materials. a) Six branches of the phonon dispersion relation in graphene, and the 2D Raman band (iTO phonon resonance) with 2.41 eV laser energy for graphene of different layers and highly ordered pyrolytic graphite (HOPG). Reproduced with permission.^[^ ^83^ ^]^ Copyright 2009, Elsevier B.V. b) SEM images with different *V*_aac_ for graphene flake with a variable thickness on SiO_2_/Si substrate. Reproduced with permission.^[^ ^89^ ^]^ Copyright 2012, John Wiley & Sons, Ltd. c) Metastable defects found in HRTEM, including Stone–Wales (SW) defect (left‐up), Reconstructed vacancy (left‐bottom), and defects consist of three pentagons and three heptagons (right). Reproduced with permission.^[^ ^92^ ^]^ Copyright 2008, American Chemical Society. d) STM spectra with different distances from point defects and tomographs of graphene on Rh substrate with tens of atomic‐scale defects and a single carbon vacancy. Reproduced with permission.^[^ ^93^ ^]^ Copyright 2016, American Physical Society. e) STM image (top) of N‐doped graphene, set point 50 pA at 1.0 V, and constant‐height AFM image (bottom) of the same location at −20 pm about STM set point. Reproduced with permission.^[^ ^94^ ^]^ Copyright 2016, American Physical Society. f) IR spectra demonstrate the functional groups of p‐G (A), GO (B), and rGO (C). Reproduced with permission.^[^ ^23b^ ^]^ Copyright 2011, Elsevier B.V. g) UV–vis absorption spectra demonstrating the red shift during reduction of GO, where peak positions and heating time versus absorbance increase are zoomed in. Reproduced with permission.^[^ ^99^ ^]^ Copyright 2020, American Chemical Society. h) High‐resolution C 1s XPS spectra of GO (left) and delphinium root extract reduced GO (right). Reproduced with permission.^[^ ^50^ ^]^ Copyright 2020, Wiley‐VCH. i) XRD spectra of GO and rGO. Reproduced with permission.^[^ ^35^ ^]^ Copyright 2019, Elsevier Ltd.

Raman spectroscopy could measure the layer of graphene and detect the defects. The basic principle of Raman spectroscopy detection is based on the lattice vibration of phonon.^[^ ^18^, ^31^ ^]^ The Raman scattering involves three steps: 1) photon excites electron vertical transition, resulting in electron–hole pair, 2) electrons or holes are scattered by phonons, and 3) scattered electron–hole recombination produces scattered photons. Considering the conversion of momentum and energy during phonon dispersion of graphene in the high symmetry ГM and ГK directions of reciprocal space, six phonon dispersion curves could be derived and applied in calculating the Raman spectrum (**Figure** **2**a). For instance, the 2D band Raman spectrum is derived from a second‐order process involving two iTO phonons near the K point. With more layers, the 2D‐band in the Raman spectrum is wider, especially high‐ordered pyrolytic graphite shows two peaks of the 2D‐band (Figure 2a).^[^ ^83^ ^]^ In the Raman spectrum of graphene, three major bands^[^ ^31^, ^35^ ^]^ exist with *E*_laser_ = 2.414 eV; the D band is located around 1350 cm^−1^, corresponding to the graphene defects, the in‐plane vibration of sp^2^ carbon atoms matches with G‐band located around 1580 cm^−1^, and 2D‐band (G′‐band) appears at 2700 cm^−1^ induced by stacking orders (second‐order dispersive Raman feature).^[^ ^84^ ^]^ The defects in graphene can be quantified with *I*_D_/*I*_G_. Increasing *I*_D_/*I*_G_ demonstrates higher defect density for regions with low defect density. For regions with a high defect density of amorphous carbon structures, decreasing *I*_D_/*I*_G_ corresponds to higher defect density.^[^ ^18^ ^]^ The reduction effect can be studied based on *I*_D_/*I*_G_, which increases with an increased degree of reduction; for instance, GO = 0.96, partially reduced GO = 1.12, and rGO = 1.6 have been reported.^[^ ^46b^ ^]^ As Raman spectroscopy has accuracy limitations, Sandro's group developed a tip‐enhanced Raman spectroscopy (TERS) to overcome the diffraction limit of confocal Raman spectroscopy.^[^ ^85^ ^]^ According to their results, TERS could detect individual point defects with higher resolution. This could be a potential detection method for detecting the point defects of functionalized graphene for biomedical applications. In terms of the thickness measurement, generally, monolayer graphene shows a lower G band intensity, whilst bilayer or multilayer graphene possesses a much wider and upshifted 2D band than monolayer graphene.^[^ ^18^ ^]^ Though providing nondestructive imaging for graphene, the thickness measurement by Raman spectrum is limited, where more accurate results are obtained from high‐quality graphene with few layers (*N* < 4) compared with defective or thick graphene sheets.^[^ ^86^ ^]^ As defective or thick graphene would also be applied in the biomedical field, other techniques to measure thickness are introduced in the following paragraphs.

SEM, TEM, and STM can be applied to measure the lateral size and thickness and detect defects in graphene. Though with similar functions, the mechanism and characterization results are different. SEM is a powerful technique to observe the thickness of graphene samples^[^ ^31^, ^87^ ^]^ based on the discretely distributed secondary electron intensity, where the contrast in images represents thickness difference.^[^ ^88^ ^]^ The primary electron acceleration voltage (*V*_acc_) is a crucial parameter in SEM;^[^ ^89^ ^]^ with high *V*_aac_, fewer secondary electrons emit from the graphene flakes than from the SiO_2_ substrate; thus, the intensity contrast is small (Figure 2b). On the contrary, there could be more secondary electrons from graphene flakes with low *V*_aac_. Although abnormal contrast with *V*_acc_ = 2 kV (Figure 2b) could be avoided by coating Au on graphene to remove positive charges,^[^ ^89^ ^]^ the Au removal would be another problem if the samples must be recycled. *V*_acc_ = 1 kV is recommended as a suitable setting for the thickness detection of graphene materials. TEM takes advantage of high resolution based on high‐energy electron penetration,^[^ ^18^, ^27^ ^]^ from which the details of graphene can be observed, and a clear atomic pattern can be seen after the Fourier transform.^[^ ^90^ ^]^ Generally, traditional TEM can be classified as bright‐field and dark‐field.^[^ ^91^ ^]^ Bright‐field TEM detects the number of electrons penetrated.

More electrons could pass through pixels with no atoms and then be detected by the detector; the dark regions correspond to atoms, and the bright regions demonstrate the gaps. Dark‐field TEM is more likely to be used in graphene analysis, where the positions of atoms can be determined by detecting the scattered electrons.^[^ ^90^, ^91^ ^]^ More electrons are scattered for a heavier and thicker sample; thus, bright regions correspond to atoms; on the contrary, dark regions represent the gap due to less scattering of electrons. Gass et al. detected atomic lattice in high‐angle annular dark‐field images with SEM.^[^ ^91^ ^]^ Similarly, atomic lattice and defects were reported to be observed with aberration–correction in combination with a monochromator according to Meyer et al.^[^ ^92^ ^]^ where resolution as high as 1 Å was achieved at an acceleration voltage of only 80 kV (Figure 2c). Recent research pays intense attention to improving imaging resolution to detect individual atoms automatically. Based on high‐resolution transmission electron microscopy (HRTEM), Vestergaard's team examined and developed a method for automatically determining variations in the atomic structure of graphene.^[^ ^90^ ^]^ STM provides higher resolution images compared with SEM and TEM.^[^ ^93^ ^]^ The basic principle is to use the electronic tunneling effect.^[^ ^1^ ^]^ If two electrodes are very close and a small voltage is applied, a tunneling current will be generated at the probe's location. The distance between the probe and the sample surface is sensitive to the tunneling current. When the probe scans the sample surface at a set position, the distance between the probe and the sample surface changes due to the roughness of the surface, and the tunneling current also changes, which then provides a difference in the STM spectra (Figure 2d).^[^ ^93^ ^]^ The arrangement of atoms on the sample's surface can be obtained by scanning the sample's surface with the probe and recording the penetrating current at each position. Therefore, STM is useful for studying the atomic properties of conductive sample surfaces. Zhang's group obtained an STM topographic image of graphene on an Rh substrate with tens of atomic‐scale defects and a single carbon vacancy of graphene (Figure 2d).^[^ ^93^ ^]^ Considering similarities between STM, TEM, and SEM, the difference in resolution, working atmosphere, sample destruction, and detection depth of these structural detection techniques are summarized in **Table** **1** to provide an overview of the effective selection of characterization methods in graphene analysis.

**Table 1:** Comparison between three typical structural detection techniques

Method	Ability	Working atmosphere	Sample destruction	Detecting depth
STM[93]	Atoms can be directly observed; with a horizontal resolution of 0.1 nm and a vertical resolution of 0.01 nm	Various environments, such as the atmosphere, ultrahigh vacuum, or liquids (including insulating liquids and electrolytes)	None	1–2 atoms (cannot detect deep information nor directly observe the insulator)
TEM[90, 91]	Horizontal point resolution: 0.3–0.5 nm; horizontal lattice resolution: 0.1– 0.2 nm; vertical resolution: none	High vacuum	Moderate	Equals to the sample thickness (<100 nm)
SEM[88, 89]	Lateral resolution: 1–3 nm, longitudinal resolution: low	High vacuum	Minor	1 µm

AFM can be applied to measure the lateral size and thickness and detect defects. The imaging mechanism of AFM is based on detecting variations in the reflected laser induced by the cantilever movements when scanning the samples, where the cantilever movements are affected by the vdW between the pin and sample. AFM takes advantage of high resolution, the magnification of an optical microscope is generally not more than 1000 times; the magnification limit of an electron microscope is 1 million times, while the magnification of AFM can be up to 1 billion times.^[^ ^18^ ^]^ As shown in Figure 2e, the resolution of images from AFM is better than from STM.^[^ ^94^ ^]^ Six imaging modes could be applied with AFM for different imaging demands, as the details are beyond the scope of this review; readers interested in that are directed to refer to the recent review.^[^ ^95^ ^]^ For defects detection, due to the atomic resolution of imaging that could be provided, both point and line defects can be directly observed from noncontact AFM imaging.^[^ ^96^ ^]^ In terms of the lateral size measurement, AFM could scan samples with step size; thus, the lateral size of graphene could be easily measured by choosing the appropriate direction.^[^ ^96^ ^]^ However, the thickness calculation of SLG was inaccurate (varied from 0.4 to 1.7 nm) if tapping mode AFM imaging with free amplitude of cantilever oscillation that is directly affected by nanomaterial–tip interactions without optimization.^[^ ^97^ ^]^ Shearer et al. proposed an accurate PeakForce tapping (PFT) mode AFM imaging to measure the thickness of graphene based on the hypothesis that pressure (peak force) determines the accuracy of measurement.^[^ ^86^ ^]^ With the PFT mode, the measure of SLG thickness was found to be accurate (0.43 nm) when increasing the peak force from 1 to 10 nN. As AFM may cause damage to the crystal lattice during measurement,^[^ ^89^ ^]^ it is probably that the PFT mode AFM imaging would be more convenient and cause less damage if the interface with a machine learning algorithm trained from interaction force, adhesion and topography dataset, and automatically determine the suitable force applied for regions in graphene sheets with variation in thickness.

##### Functional Groups

Functional groups on the surface of graphene benefit from working as a bridge to proceed with functionalization methods targeting biomedical applications. Fourier transform infrared (FT‐IR), ultraviolet–visible (UV–vis), XPS, and X‐ray diffraction (XRD) are typical characterization techniques for functional group detection of graphene. FT‐IR is widely utilized to analyze the bond types in molecules^[^ ^1^ ^]^ as the infrared absorption spectrum is a typical molecular absorption spectrum,^[^ ^27^ ^]^ and the types of graphene materials can be distinguished based on the bond type and absorbance.^[^ ^23b^ ^]^ When the sample is irradiated by infrared light with continuous frequency changes, the molecule absorbs energy from radiation with a specific frequency. Its vibration or rotational motion induces a net change of dipole moment. This further results in the transition of molecular vibration and rotational energy level from the ground state to the excited state, weakening the transmitted light intensity corresponding to these absorption regions. The FT‐IR spectrum is obtained by recording the curve between the percentage transmittance of infrared light and wavenumber or wavelength after Fourier transform.^[^ ^31^ ^]^ Some corresponding standard relationships exist between wavelengths in the FT‐IR spectrum and bond types.^[^ ^35^, ^50^ ^]^ For instance, 1620 nm is attributed to the aromatic C=C bond, 1060 nm corresponds to the C—O stretching vibration mode in the alkoxy group, 1170 nm originates from the epoxy C—O stretching peak, 1400 nm arises from the C—OH carboxyl group, 1740 nm is attributed to the C=O stretching mode in the carboxyl group, 2930 nm is assigned to C—H, and 3420 nm is for the O—H groups (Figure 2f).^[^ ^23b^ ^]^ As shown in Figure 2f, the absorbance signals of bond types in p‐G, GO, and rGO are different; for example, rGO exhibits the highest absorbance signal of all bond types, from which the types of graphene materials can be identified. UV–vis or visible irradiation brings more energy to molecules than IR.^[^ ^18^ ^]^

UV–vis induces electron transitions in molecules, while IR causes vibrations and rotations in molecules. UV–vis absorption spectrum is generated based on the valence electron transition after the molecule (or ion) absorbs UV or visible light (usually 200–800 nm).^[^ ^1^, ^35^ ^]^ The UV–vis spectrum presents a wideband as the energy level transition between electrons is always accompanied by the transition between vibrational and rotational energy levels.^[^ ^18^, ^50^ ^]^ There is also a matchup between bond types and electron transition modes, such as *π*→π* transitions from the aromatic C=C bond and the n→π* transition originates from the C=O bond.^[^ ^98^ ^]^ Additionally, GO, partially reduced GO and rGO can be distinguished based on the peak shifts in the UV–vis spectrum. For example, 220 and 310 nm correspond to specified transitions of *π*–π* (C=C) and n–π* (C=O) in GO.^[^ ^46b^ ^]^ Additionally, red shifts of the absorbance peaks appeared with an increased degree of reduction, for instance, from 226 to 258 nm after thermal treatment (reduction from GO to rGO) at 100 °C for 280 h from which different graphene materials could be identified (Figure 2g).^[^ ^99^ ^]^

XPS radiates samples with X‐rays so that the inner or valence electrons of atoms or molecules are excited and ejected.^[^ ^1^ ^]^ Electrons excited by photons are called photoelectrons, and the intensity (or counts) or corresponding energy of these photoelectrons are needed to derive an XPS spectrum.^[^ ^31^ ^]^ Usually, peak positions in XPS are related to different functional groups. The functional groups in the samples can be ensured to identify further the type of graphene materials (Figure 2h).^[^ ^50^ ^]^ For example, GO, and rGO can be distinguished based on XPS, where the intensity of sp^2^ carbons in aromatic rings (C=C, 284.5 eV) exhibits no change. By contrast, the intensity of carbonyl (C=O, 287.7 eV) peaks significantly decreases in the case of rGO.^[^ ^41^, ^50^ ^]^

XRD utilizes diffraction and scattering of X‐ray photons during irradiation to derive information, including crystal shapes, phase, and functional groups of samples.^[^ ^18^ ^]^ According to the Bragg equation, 2*d_hkl_* sin*θ* = *nλ*, where *d_nkl_* is the grating constant, is inversely proportional to *θ*; the larger the crystal plane spacing, the smaller the diffraction angle.^[^ ^1^ ^]^ Each crystalline substance has specific structural parameters, including lattice type, cell size, etc. Thus, the number, position, and intensity of the diffraction peaks are characteristic of each substance, like a person's fingerprint.^[^ ^31^ ^]^ Although there are thousands of substances, there are almost no two substances whose diffraction spectra are the same, so a qualitative analysis of the phase of the substance can be carried out.^[^ ^50^ ^]^ The XRD spectra of GO and rGO showed in Figure 2i are a good instance; there is an increase of d_nkl_ in GO due to the oxygen moieties between graphene layers. Thus, the peak at 12.2° of GOs shifts to 26.64° of the rGO, which brings an obvious difference in the XRD spectra between the two samples.^[^ ^35^ ^]^

##### Dispersion Behaviors

Dispersion behaviors include concentration, stability, and decomposition/aggregation of graphene materials in solvents, which influence the functionalization and treatment efficiency, biodegradation, and transportation of graphene‐based products. UV–vis is generally applied to analyze the concentration; stability can be measured by Zeta potential, while decomposition or aggregation of graphene materials can be evaluated by dynamic light scattering (DLS). In the last part, UV–vis is introduced to detect functional groups; while increasing UV wavelength and achieving a maximum wavelength >500 nm, the adsorption curve would be flat, and optical density is dependent on concentration.^[^ ^71a^ ^]^ The graphene concentration could be calculated with Lambert–Beer law (*A* = *l* × *α* × *C*) from the UV–vis spectrum, where *A* denotes UV absorption in graphene, *l* is the optical path length in cm, *α* represents the absorption coefficient, and *C* is the concentration of graphene.^[^ ^100^ ^]^ It is noteworthy that *α* depends on the lateral size, layers, and the number of functional groups of graphene; thus, it needs to be modified when applied to examine different samples.^[^ ^101^ ^]^ It could be determined either from the gradient of an absorbance/length versus concentration plot^[^ ^100^ ^]^ or using the literature values.^[^ ^71a^ ^]^

The stability of graphene materials is evaluated based on Zeta potential in solvents. When graphene is immersed in a solvent, as the solid surface generally obtains a certain surface charge density, and the liquid has dissolved ions and a high dielectric constant, the interface will be charged.^[^ ^74^ ^]^ For instance, graphene in water is typically negatively charged on the surface, and the close interface layer would obtain opposite‐charged ions. There exists a diffusion layer outer the interface layer, where solvent particles and dissolved ions move in this layer. Furthermore, a shear plane in this layer is chosen, ions inside the boundary move with the particle, but ions outside the boundary do not move with the particle. The potential at the shear plane is defined as Zeta potential, where the value generally <10 mV demonstrates unstable, 10–30 mV indicates slightly to moderately stable, 30–60 mV represents good stability, and samples with the Zeta potential >60 mV exhibit excellent stability.^[^ ^71^, ^102^ ^]^ It is noteworthy that long‐term stability is important for the commercial application of graphene solvents; thus, a routine Zeta potential measurement is necessary for long‐term evaluation.

DLS is generally applied to measure the size of graphene sheets in solvents to evaluate whether they are decomposed or aggregated to smaller or larger clusters during dispersion.^[^ ^16^, ^46^ ^]^ The basic principle of DLS for size measurement is monitoring the temporal change in scattering intensity as the particles move under Brownian motion.^[^ ^71a^ ^]^ It is noteworthy that the lateral and rotational diffusion parameters need to be considered when dealing with the raw data, as graphene sheets are generally flake instead of spherical particles. For instance, the lateral size of GO was determined from the translational diffusion coefficient with depolarized DLS using a model for circular, infinitely thin disks.^[^ ^103^ ^]^ The radius of GO was measured as 157 ± 22 nm based on the spherical model, which is smaller than the real value, while the radius provided with their model was more accurate (194 nm). Therefore, DLS with a suitable model would be more accurate and beneficial to the long‐term evaluation of biodegradation behavior.

#### 2.3.2 Functional Analysis

##### Intrinsic Properties

The synthesized graphene materials must be applied in biomedical fields; therefore, electrical conductivity for biosensing, laser absorption for photothermal therapy (PTT), mechanical properties for tissue engineering, ferromagnetic property for magnetic field drive, and thermal stability of graphene materials under different environments need to be well evaluated. The analysis of these properties is flexible and with wide alternatives; thus, several typical methods are introduced here. For electrical conductivity analysis, cyclic voltammograms (CV) measurements and resistivity changes on gate voltage were widely utilized.^[^ ^58^, ^104^ ^]^ The shape of CV reflects the detection speed, intensity, and reproductivity of biomolecules and evaluates the working life of graphene‐based biosensors.^[^ ^105^ ^]^ Sensogram could provide information on resistance variation with an increase in dose on the biosensor, demonstrating the sensitivity and limit of detection for the graphene‐based biosensor.^[^ ^10^ ^]^ Laser absorption could be evaluated by observing the temperature rises induced by the NIR laser.^[^ ^72^, ^106^ ^]^ For instance, an 808 nm NIR was applied on GO and rGO solvents, and the temperature rise varied with time, demonstrating the photothermal therapy efficiency of graphene materials and assisting with determining suitable irradiation duration during therapy.^[^ ^76^ ^]^ Mechanical properties like stress, strain, and Young's modulus could be analyzed by the Zwick machine or AFM.^[^ ^107^ ^]^

**Figure 3:**
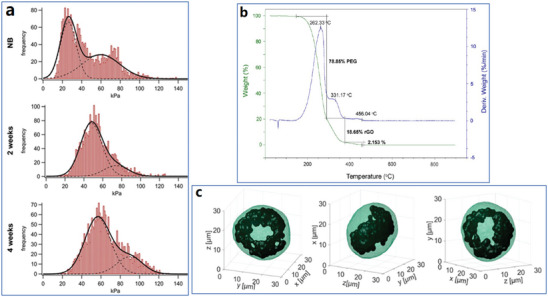
Typical functional analysis techniques were utilized to examine the properties of graphene materials. a) Stiffness distribution based on indentation‐type AFM measurements of murine cartilage samples from newborn, two weeks, and four‐week mice. Reproduced with permission.^[^ ^108^ ^]^ Copyright 2015, The Authors. Published by Elsevier B.V. b) TGA curve of PEG–rGO. Reproduced with permission.^[^ ^74^ ^]^ Copyright 2016, American Chemical Society. c) Reconstructed images based on FCM data and AM‐TFC algorithms for research of biodistribution of GO in NIH‐3T3 cell (48 h after GO treatment). Reproduced with permission.^[^ ^112^ ^]^ Copyright 2021, American Chemical Society.

Zwick machine provides information on the mechanical properties based on the deformation and stress of samples while providing continuously varied force. The results benefit from evaluating the ability to apply graphene materials in tissue engineering.^[^ ^107b^ ^]^ As there is a very weak force (repulsion or van der Waals) between the atoms at the top probe of AFM and those on the sample's surface, indentation‐type AFM measurements can be utilized to analyze the mechanical properties.^[^ ^1^, ^89^ ^]^ Prein et al. reported that the force required to push the tip into the sample is a quadratic function of the indentation depth.^[^ ^108^ ^]^ They presented cartilage's stiffness distribution at different timelines by providing a constant force and measuring the maximum hitting frequency (**Figure** **3**a). It is supposed that indentation‐type AFM measurements can be applied to measure the mechanical properties of graphene sheets and bone cell lines grown on graphene substrates. However, indentation‐type AFM measurements may destroy the samples, and it is necessary to consider them carefully before using this technique. Magnetic hysteresis evaluated Ferromagnetic properties, and superparamagnetic or antiferromagnetic interactions could be judged from the hysteresis features.^[^ ^22^, ^109^ ^]^ The magnetic hysteresis loop demonstrates the stability and thermal conversion of graphene materials with the magnetic field, assisting in evaluating the thermal treatment efficiency controlled by the magnetic field.^[^ ^22^ ^]^ At the same time, the saturation magnetization reflects the sensitivity of magnetic targeting drug delivery for superparamagnetic GO–Fe_3_O_4_ nanohybrid.^[^ ^109^ ^]^

Thermogravimetric analysis (TGA) is generally applied to evaluate the thermal stability of materials^[^ ^31^, ^35^ ^]^ and the proportion of each compound in the complex.^[^ ^25^ ^]^ The mass change and change rate in graphene materials could be accurately measured. For example, Mendonça et al. presented the TGA curve of PEG–rGO, as shown in Figure 3b, where the ratio of grafted PEG was estimated to be 78.85%.^[^ ^74^ ^]^ Recent studies tend to combine multiple biomedical applications based on graphene materials; thus, it would be essential to choose suitable functional analysis methods to evaluate graphene‐based materials flexibly.

##### Extrinsic Properties

Observing the interaction of graphene in a physiological environment, especially the interaction with cells, is important in the biomedical application of graphene. Flow cytometry (FCM) can give quantitative measurements of the cellular uptake of graphene materials^[^ ^110^ ^]^ as side scattering is correlated to cellular granularity. Also, the secretion of cytokines induced by graphene can be detected by FCM.^[^ ^111^ ^]^ Pirone et al. developed a tomographic flow cytometry (TFC) method to directly demonstrate the intracellular biodistribution of graphene materials in 2021.^[^ ^112^ ^]^ After reconstruction of the tomography with different angles and times based on AM‐TFC algorithms, 3D graphene distribution in an NIH‐3T3 cell after 48 h treatment with GO has been presented (Figure 3c). Another interesting FMC‐based imaging technique has been proposed by Nedosekin et al., who developed a photoacoustic flow cytometry technique for noninvasive detection and real‐time label‐free monitoring of circulating graphene‐based nanoparticles in vivo with mice models.^[^ ^110a^ ^]^ According to their results, the dynamics study of the circulation clearance for p‐G, functionalized graphene (f‐G), and GO was similar. However, GO (hydrophilic) circulated mainly as single flakes, whereas p‐G (hydrophobic) and f‐G (partially hydrophobic) formed multiple large clusters in circulation.

## 3 Functionalization Methods

**Figure 4:**
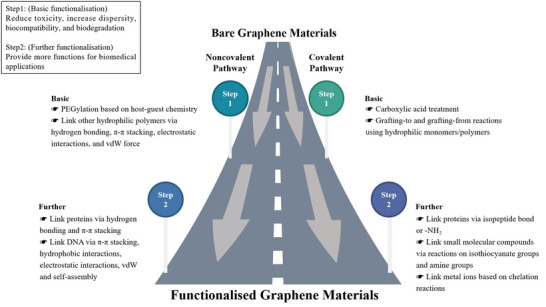
Schedule of typical functionalization methods for graphene materials.

Bare graphene materials have drawbacks when applied in vivo, such as dose‐,^[^ ^43^ ^]^ size‐,^[^ ^113^ ^]^ time^[^ ^114^ ^]^‐related toxicity, poor dispersity,^[^ ^115^ ^]^ low biocompatibility,^[^ ^16^ ^]^ and poor biodegradation.^[^ ^12a^ ^]^ To improve dispersion and stability, prolong circulation, and reduce cytotoxicity, basic functionalization based on noncovalent and covalent methods is applied by utilizing hydrophilic polymers or carboxylic acids. Successively, further functionalization using proteins, DNA, metal nanoparticles, etc., is useful for achieving a more specific application efficiently. There are differences between noncovalent and covalent methods; in terms of the basic principle, noncovalent approaches usually rely on the stabilization effects (like hydrogen bonding interactions,^[^ ^116^ ^]^ *π*–*π* stacking,^[^ ^43^ ^]^ electrostatic interactions,^[^ ^117^ ^]^ and van der Waals forces^[^ ^3^ ^]^) of surfactants that adsorb at the graphene surface. By contrast, covalent bonds are formed when reactions occur based on the functional groups (—COOH/—NH_2_) between functionalized materials and graphene.^[^ ^115^ ^]^ In terms of features, noncovalent modification methods could preserve electrical conductivity and present a large graphene surface,^[^ ^37^ ^]^ while covalent methods provide better stability and robust mechanical properties.^[^ ^118^ ^]^ This section discusses the basic and further functionalization from both covalent and noncovalent methods, as shown in **Figure** **4**.

### 3.1 Basic Functionalization

Bare p‐G has few functional groups^[^ ^115^ ^]^ and a hydrophobic surface,^[^ ^16^ ^]^ while bare GO or rGO obtain sharp edges^[^ ^119^ ^]^ and a negative charge surface.^[^ ^120^ ^]^ Also, bare graphene materials are synthesized with a few size or thickness control,^[^ ^111a^ ^]^ thus causing dose‐,^[^ ^43^ ^]^ size‐,^[^ ^113^ ^]^ time^[^ ^114^ ^]^‐related toxicity, poor dispersity,^[^ ^115^ ^]^ low biocompatibility,^[^ ^16^ ^]^ and poor biodegradation.^[^ ^12a^ ^]^ Therefore, basic functionalization methods that utilize hydrophilic polymers or carboxylic acids to modify the properties of bare graphene are needed.^[^ ^1^, ^121^ ^]^ After functionalization, the graphene materials obtain better dispersity and stability, less toxicity, and can support subsequent further functionalization with other molecules.

#### 3.1.1 Noncovalent Functionalization

During noncovalent functionalization methods, mixing the materials at a suitable temperature and applying ultrasonication are needed. A large number of materials were utilized to noncovalently functionalize graphene via hydrogen bonding interactions,^[^ ^116^ ^]^ *π*–*π* stacking,^[^ ^43^ ^]^ electrostatic interactions,^[^ ^117^ ^]^ and van der Waals force.^[^ ^3^ ^]^ PEGylation of graphene is the most common and well‐established method.^[^ ^74^ ^]^ As reported by several groups, rGO tends to agglomerate irreversibly or even to restack into graphite through *π*–*π* stacking interactions.^[^ ^43^ ^]^ At the same time, the noncovalent bonding of PEG with rGO could largely improve the dispersity and stability and inhibit aggregation, which may be attributed to the increasing steric hindrance.^[^ ^122^ ^]^ The rGO toxicity was also pacified by PEGylation, with a concentration of less than 10 µg mL^−1^, and there was almost no toxicity without reactive oxygen species (ROS) generation.^[^ ^23^, ^74^ ^]^ Additionally, amino‐terminated PEG could act as a bridge to link functional molecules by amido bond further.^[^ ^43^ ^]^

Apart from PEG, other hydrophilic polymers have been reported to improve dispersity and stability and reduce toxicity. Several groups utilized pyrene‐terminated side‐chain liquid crystalline polymers^[^ ^14^ ^]^ and polystyrene sulfonate (PSS)^[^ ^102b^ ^]^ to functionalize GO via *π*–*π* interactions. Natural polymer dextran could functionalize GO based on hydrogen bonding and *π*–*π* stacking.^[^ ^109^, ^123^ ^]^ Moreover, PVA generally interacts with GO based on hydrogen bonding.^[^ ^116^, ^124^ ^]^ Also, a few groups functionalized GO with cationic polymers like polyethyleneimine (PEI),^[^ ^117^ ^]^ polypropylene imine (PPI),^[^ ^125^ ^]^ and poly(amidoamine) (PAMAM)^[^ ^118^ ^]^ based on electrostatic interaction. Additionally, physical adsorption was applied in earlier studies; polyoxyethylene sorbitan laurate (TWEEN 20) was also physically adsorbed on rGO via the van der Waals force.^[^ ^126^ ^]^ Similarly, Fan et al. examined the functionalization of FLG with chitosan (a type of polysaccharide) in an acid solution to obtain better biocompatibility^[^ ^62^ ^]^ via physical adsorption.

Noncovalent functionalization methods require a relatively simple process where materials and graphene interact without a chemical reaction. Thus, the unique properties of graphene are preserved while dispersity, stability, and toxicity are modified. However, noncovalent interaction is not strong enough to undergo intense extrinsic stimuli like NIR laser irradiation or high‐temperature exfoliation. Also, noncovalent interaction cannot significantly change electronic transport properties if used in biosensing.^[^ ^127^ ^]^

#### 3.1.2 Covalent Functionalization

Covalent functionalization methods are adapted to obtain a stronger interaction between graphene and other materials, as well as enhance electronic transport,^[^ ^127^ ^]^ where carboxylic acids or hydrophilic polymers are widely used. Carboxylic acid such as tetraphenyl porphyrin bearing sulfonic acid groups^[^ ^110b^ ^]^ or dilute nitric acid^[^ ^38a^ ^]^ led to the attachment of carboxyl, epoxy, and hydroxyl side groups on graphene, with which the functionalized graphene is more dispersible in the aqueous phase.^[^ ^75^ ^]^ Although carboxylic acid treatment is relatively simple, this method may introduce other active molecules through bioconjugation chemistry^[^ ^53^ ^]^ or create more defects on graphene sheets due to oxidation.^[^ ^16^ ^]^ Therefore, hydrophilic polymers are more likely to be applied.

Previous research classified graphene modification methods with polymers or poly‐carboxylic acids as grafting‐from and grafting‐to methods.^[^ ^127^, ^128^ ^]^ Grafting‐from methods consider graphene the initiation site to grow a polymer from monomers. By contrast, in grafting‐to methods, the presynthesized polymers are end‐tethered to the graphene surface.^[^ ^127^ ^]^ For grafting‐from methods, various polymerization methods were utilized, including 1) atom transfer radical polymerization of polystyrene (PS), poly(tertbutyl acrylate),^[^ ^128^ ^]^4 poly(methyl methacrylate) (PMMA),^[^ ^62^ ^]^ 2) condensation of poly(glycerol sebacate) (PGS) to graphene,^[^ ^107b^ ^]^ 3) attaching PEI to GO based on ring‐opening polymerization,^[^ ^125^ ^]^ 4) the direct electrophilic substitution of polyether ketones to graphene,^[^ ^128^ ^]^ and 5) Ziegler Natta polymerization of polypropylene (PP) to GO based on catalysts.^[^ ^28^, ^128^ ^]^ In terms of grafting‐to methods, different kinds of chemical reactions were used to provide strong covalent interactions, including 1) esterification/amidation reactions of carboxylic groups in GO/rGO with hydroxyl or amine groups in the polymers of PEG, PVA, and PEI,^[^ ^129^ ^]^ 2) nitrene cycloaddition for the functionalization of p‐G/rGO with single molecules of polymers like PS, PEG,^[^ ^13^ ^]^ 3) cross‐linking by amine‐induced ring‐opening/esterification/nucleophilic substitution to provide functionalized graphene/GO with polymers of epoxy or poly(N‐isopropylacrylamide),^[^ ^16^, ^128^ ^]^ 4) opening of maleic rings in maleic acid,^[^ ^128^ ^]^ and 5) radical grafting of PMMA to rGO by phase transfer.^[^ ^59^ ^]^ It is not easy to precisely choose a suitable covalent functionalization method among manifold reactions. In **Table** **2**, the advantages and drawbacks of grafting‐from and grafting‐to methods have been summarized.

**Table 2:** Comparison between grafting‐from and grafting‐to methods

	Advantages	Drawbacks
Grafting‐from methods	1) Not limited by steric hindrance nor by the presence of large graphitic sheets; thus, a relatively high molecular weight polymer was obtained.[62, 107] 2) The polymer/initiator ratio can be varied to control the final composition of graphene materials,[128] and generally, the grafting ratio is higher than the grafting‐to method.[127] 3) The grafting density can be controlled by setting the location at either basal planes or edges of graphene.[128]	1) Some polymers are not suitable for polymerization or with relatively low efficiency on graphene sheets.[13, 28] 2) The relative amount of graphene in the compound is lower than the grafting‐to method, and the molecular weight or polymer distribution is hard to be controlled precisely.[128]
Grafting‐to methods	1) The percentage of surface coverage is higher in the grafting‐to approach than in the grafting‐from method, which may be beneficial in cases where interaction between graphene and cells needs to be avoided.[127] 2) Allows the combination of a wide variety of polymers with graphene, including those that cannot be polymerized from the graphene surface.[128, 129]	1) The steric hindrance induced by huge graphene sheets may influence the degree of functionalization.[6] 2) The relative amount of graphene in the compound is hard to control externally, as the system determines it in terms of tactic sequences and steric hindrance.[128]

### 3.2 Further Functionalization

Basic functionalization could only provide limited benefits for biomedical applications; thus, the successive further functionalization methods provide more functions to graphene materials by utilizing proteins,^[^ ^130^ ^]^ nucleosides/nucleotides^[^ ^131^ ^]^ or metal ions.^[^ ^6^ ^]^ After functionalization, the graphene materials exhibited more unique properties and could be used in biomedical imaging, drug/gene delivery, tissue engineering, biosensing, and immunotherapy.

#### 3.2.1 Noncovalent Functionalization

Similarly, noncovalent functionalization includes hydrogen bonding, *π*–*π* stacking, electrostatic and hydrophobic interactions, van der Waals forces, and interaction based on self‐assembly. The functionalization of graphene materials with proteins,^[^ ^15^ ^]^ doxorubicin (Dox),^[^ ^98^ ^]^ Pluronic F38 (F38),^[^ ^109^ ^]^ Tween 80 (T80), and maltodextrin (MD)^[^ ^7^ ^]^ generally involves hydrogen bonding and *π*–*π* stacking. The binding of DNA chains to GO sheets was found to occur through *π*–*π* stacking and hydrophobic interactions between the bases of DNA and graphitic domains of GO.^[^ ^132^ ^]^ Electrostatic/hydrogen bonding interactions were also found between the prime amines of bases and the oxygen‐containing groups of GO.^[^ ^131^, ^133^ ^]^ Furthermore, the functionalization of several proteins utilized even more interactions. The adsorption of steroid hormones on GO nanosheets is a good example, where hydrophobic and electrostatic interactions present in long‐range up to 10 nm and short ranges (2–5 nm) interactions include *π*–*π* stacking, van der Waals forces, and hydrogen bonding.^[^ ^134^ ^]^ Several groups adapted self‐assembly to connect DNA,^[^ ^131^ ^]^ SS‐GrBP5 mutant,^[^ ^10^ ^]^ polyoxyethylene sorbitan laurate (TWEEN 20),^[^ ^126^ ^]^ and photoreceptor proteins^[^ ^17^ ^]^ to graphene.

It is noticed that the noncovalent functionalization of proteins, nucleosides/nucleotides is always based on the synergistic effect of multiple interactions related to the distance between molecules and graphene. Noncovalent interaction is useful when the controlled release of drugs or DNA is needed. However, such connections are not strong enough to bind with the functionalization agents that need a stable connection. Hence, covalent interactions are introduced to provide stronger interactions.

#### 3.2.2 Covalent Functionalization

Stronger interactions provided by covalent functionalization are required if applied in PTT, biomedical imaging, or tissue engineering. Chemical reactions also induce covalent interactions. This part introduces the categories of interactions of proteins, small molecular compounds, and metal ions. In terms of connecting proteins, forming an isopeptide bond between graphene and SpyTag,^[^ ^57a^ ^]^ attaching BLUF proteins through the reactive N‐hydroxysuccinimide ester groups,^[^ ^17^ ^]^ and bonding enzymes, SBA‐15 antibody through —NH_2_ and reactive functional groups on graphene^[^ ^135^ ^]^ are reported. The covalent functionalization of proteins can support specific graphene‐complex targeting to target cells or tissues. For covalent bonding of graphene and small molecular compounds, reacting with thionyl chloride followed by the reaction with octadecylamine on graphene,^[^ ^136^ ^]^ chemical coupling of isothiocyanate groups and amine groups to link rhodamine B isothiocyanate,^[^ ^6^ ^]^ using carbodiimide‐catalyzed amide formation to bind Cypate^[^ ^8^ ^]^ are few examples. These small molecular compounds with a stable connection to graphene can be applied in PTT and fluorescence imaging. As for metal ions, the graphene sheet chelated metal ions, including Cu^2+[^ ^6^ ^]^ and Gd(III),^[^ ^132^ ^]^ formed a complex system analogous to a metal compound with an expanded planar ligand. These complexes could be applied as imaging contrast agents or enhancing PTT. However, chemical reactions between graphene and nucleosides/nucleotides for covalent functionalization are not fully understood. Further covalent functionalization aims to provide more functions to graphene for specific biomedical applications. Generally, interactions between proteins, specific drugs, metal ions, and graphene sheets by this method are quite strong. Due to low concentration, these proteins, specific drugs, and metal ions induce negligible influence on graphene's basic properties.

## 4 Toxicity and Biocompatibility

The toxicity and biocompatibility of graphene materials need to be carefully evaluated prior to any biomedical applications, and it would be optimal to conform to preclinical safety pharmacology guidelines of regulatory agencies such as the Food and Drug Administration (FDA), International Conference on Harmonization (ICH), and the European Medicines Agency (EMA).^[^ ^42^ ^]^ Bare graphene materials induce cytotoxicity in vitro and physiological damage in vivo, as reported by a few studies.^[^ ^79^, ^137^ ^]^ Therefore, a clear understanding of the interaction mechanism between p‐G, GO, rGO, and various cells or biomolecules in vivo and in vitro is crucial to avoid undesired severe side effects during application.

This section introduces the interaction mechanisms, followed by five routes of cytotoxicity induced by graphene materials in vitro in Section 4.1. Then, the in vivo distribution and histological changes after the administration of graphene materials in vivo have been interpreted. Cells discussed in this part cover macrophages, cell lines from organs, and immune cells. Furthermore, several potential solutions to mitigate toxicity have been proposed based on the mechanism of toxicity and inflammatory response induced by graphene materials in vivo and in vitro. In the next section of biocompatibility (Section 4.2), biocompatible evaluation (mainly hemocompatibility) of graphene materials in the physiological environment is elucidated, as well as interactions and influence on red blood cells (RBCs), platelets, and hemoglobin are involved. Furthermore, the main barrier graphene materials face for commercial biomedical application and administration from FDA/ICH/EMA approval are introduced in Section 4.3.

### 4.1 Toxicity Induced by Graphene Materials

#### 4.1.1 In Vitro Cytotoxicity

In vitro tests treat various cell lines with graphene materials outside the body to provide a basic idea of whether graphene materials cause severe damage to cells. MTT,^[^ ^122^ ^]^ CKK8,^[^ ^102b^ ^]^ and annexin V assay^[^ ^138^ ^]^ are generally used to measure cell viability, especially cytotoxicity induced by gene mutation tested by ingenuity pathway analysis (IPA).^[^ ^139^ ^]^ Considering various cytokine secretion, mRNA expression, and metabolites involved in the study, omics approaches combined with machine learning would be a high‐efficiency choice for analysis when carrying out all these three nanosafety investigations. In this section, the interactions between cells and graphene materials are first introduced. The five routes inducing cytotoxicity, including ROS generation,^[^ ^140^ ^]^ mitochondrial membrane potential (MMP) depletion,^[^ ^141^ ^]^ cell starvation,^[^ ^16^ ^]^ physical damages,^[^ ^49a^ ^]^ and gene toxicity, have been interpreted.^[^ ^139^ ^]^

##### Interactions between Cells and Graphene Materials (Macrophages as an Example)

Before introducing cytotoxicity, it is important to understand the interaction between cells and graphene materials, where researchers widely utilized macrophages to study the interaction.^[^ ^111^, ^140^ ^]^ Macrophages are vital in the phagocytosis of foreign bodies and in alerting the rest of the immune system against invaders to facilitate innate or adaptive immune responses.^[^ ^1^ ^]^ The bare graphene materials are first divided as p‐G, GO, and rGO, the uptake and clearance of macrophages for these materials are described, their side effects on macrophages are introduced, and finally, potential research areas that need further investigation are proposed.

**Figure 5:**
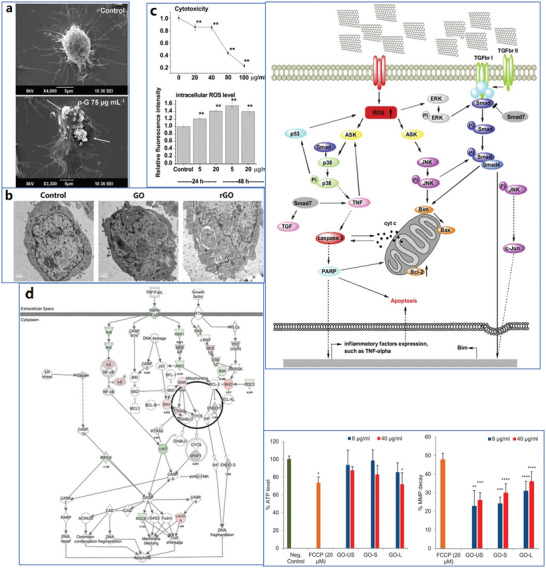
Interaction between graphene materials and cells and typical cytotoxicity pathways induced by graphene materials. a) SEM images of graphene‐treated RAW 264.7 cells of control and p‐G (75 µg mL^−1^). Reproduced with permission.^[^ ^38a^ ^]^ Copyright 2012, Wiley‐VCH. b) TEM images of interactions of GO or rGO (50 µg mL^−1^) with THP‐1 cells. Reproduced with permission.^[^ ^143^ ^]^ Copyright 2017, Elsevier B.V. c) The cell viability and intracellular ROS levels of RAW 264.7 cells treated with different concentrations of p‐G, and the schematic diagram of cell apoptosis by ROS generation induced by p‐G. Reproduced with permission.^[^ ^140^ ^]^ Copyright 2011, Elsevier Ltd. d) Mitochondria‐dependent apoptosis pathway derived from IPA analysis of RNA‐sequencing data of BEAS‐2B cells exposed to GO‐US (5 µg mL^−1^) for 28 days, and ATP level/MMP decay of cells after exposure of GO with different sizes (1–30 µm, 50 nm–2 µm, and 50–300 nm) and doses for two days. Reproduced with permission.^[^ ^139^ ^]^ Copyright 2020, Wiley‐VCH.

Regarding uptake and clearance based on macrophages for graphene materials, p‐G stays on the plasma membrane early due to its hydrophobic surface (**Figure** **5**a), resulting in an abnormal filopodial extension that immobilizes the cell.^[^ ^38a^ ^]^ After 30 days, cells would gradually internalize p‐G.^[^ ^30^ ^]^ Relatively hydrophilic GO and rGO were reported to be nonspecifically captured by macrophages of the mononuclear phagocyte system in the liver and spleen^[^ ^142^ ^]^ and THP‐1 human acute monocytic leukaemia cells (Figure 5b).^[^ ^143^ ^]^ It is noteworthy that p‐G does not damage the membranes of macrophages.^[^ ^144^ ^]^ By contrast, strong electrostatic interaction between GO sheets and membrane may lead to close interaction and provoke physical damage.^[^ ^46a^ ^]^ It was reported that graphene materials of small size could be cleared through macrophages, for example, eliminating the phagocytized FLG by alveolar macrophages through the tracheobronchial tree toward the larynx.^[^ ^12a^ ^]^ However, large‐size graphene could not be cleared.^[^ ^79^ ^]^ Therefore, size control is necessary during synthesis and functionalization to prevent graphene accumulation.

Most studies about the interaction of graphene and macrophage focus on p‐G (hydrophobic) and GO (hydrophilic), while a few focus on rGO. The potential contributing factor could be a large variation in the synthesized rGO. It is not easy to evaluate the degree of reduction in rGO quantitatively and the hydrophilicity of rGO ranges between p‐G and GO. Considering that the difference between GO and rGO has been noticed when treated on macrophages,^[^ ^143^ ^]^ it is a potential area to quantitatively evaluate the relationship between the amount of —COOH/—OH on rGO and macrophage uptake/gene transcripts.

##### Cytotoxicity Induced by Graphene Materials

Five routes induce cytotoxicity, ROS generation, MMP depletion, cell starvation, physical damage, and gene toxicity.

##### ROS Generation

ROS can induce intracellular protein inactivation (through oxidation and nitration), lipid peroxidation, mitochondrial dysfunction, and eventually apoptosis or necrosis.^[^ ^137^, ^145^ ^]^ Generally, ROS generation is caused by two pathways in mammal cell lines, including 1) a defence mechanism against foreign substances by macrophages,^[^ ^1^ ^]^ 2) lipid peroxidation in the mitochondrial respiratory chain and polyunsaturated mitochondrial membrane.^[^ ^134^ ^]^ Generation of ROS in macrophages follows route (1), where ROS generation is part of a defence mechanism against foreign substances by macrophages as well as a marker for its activation,^[^ ^1^ ^]^ and the increase of ROS level activates signaling of various routes, finally induce cytokine secretion and cell apoptosis.^[^ ^111a^ ^]^ p‐G was reported to activate mitogen‐activated protein kinase and transform growth factor‐*β* (TGF‐*β*) signaling, activating two proapoptotic members, Bim and Bax, of the Bcl‐2 protein family. Then, the activation of caspase 3 and its downstream effector proteins, such as PARP, occurs, and cell apoptosis is induced (Figure 5c).^[^ ^137^, ^140^ ^]^

Generation of ROS in other cells was through route (2); bronchial epithelial cells (BEAS‐2B) and alveolar epithelial cells (A549) were treated with GO and rGO with different sizes and functional groups.^[^ ^46a^ ^]^ Due to small lateral size and sharp corners, uptake of thermally reduced GO occurred easily, leading to less cellular viability, more ROS generation, genotoxicity, and cell death than chemically reduced GO (CRGO) and GO. Functional groups on GO provide them with a better affinity toward cell membrane, leading to an increased internalization than CRGO. Therefore, graphene with smaller sizes and more functional groups would induce more toxicity. It was also proposed that inhibitors like Nec‐1 (for A549 cells), Q‐Vd‐Oph (for BEAS‐2B cells), and N‐acetyl‐l‐cysteine (for human kidney proximal tubule epithelial cells) could reduce the damage caused by graphene to lung cells.^[^ ^46a^ ^]^

Importantly, due to the differences in organelle in these cell lines, there were differences in the routes of ROS generation by tissue cells compared with macrophages. It has been noticed that GO and rGO do not induce ROS generation in macrophages but in tissue cells.^[^ ^111^, ^143^ ^]^ It is probably due to macrophages containing very few mitochondria; thus, internalized GO and rGO would not generate ROS and has negligible cytotoxicity, while in normal tissue cells with plenty of mitochondria, ROS generation could occur via lipid peroxidation; however, further investigation is required to confirm this hypothesis.

##### MMP Depletion

MMP plays a key role in maintaining the proton gradient across the mitochondrial membrane and is essential for the electron transfer chain.^[^ ^111a^ ^]^ p‐G was found to depolarize mitochondrion, and MMP was depleted due to altering the mitochondrial membrane integrity.^[^ ^140^ ^]^ This further induces severe cell malfunctions, such as decreased ATP synthesis, more ROS generation, and the redistribution of proapoptotic mitochondrial factors.^[^ ^139^ ^]^ MMP was reported to occur in macrophages.^[^ ^137^ ^]^ However, macrophages obtain few mitochondria, and mitochondrial transports occur from other cells to macrophages. During transportation, p‐G on the membrane may interrupt the MMP. However, due to the large internalization of relatively hydrophilic GO and rGO, minor alterations in ROS and MMP were found, so GO, and rGO were less toxic than p‐G when treated with macrophages.^[^ ^46^, ^146^ ^]^ With the careful control of the size and dose of GO and rGO at 50–300 nm and <75 µg mL^−1^, respectively, nearly no toxicity was induced in macrophages.^[^ ^122^, ^139^, ^147^ ^]^ However, for other cells from the testis, epididymis, fertility,^[^ ^148^ ^]^ and kidney,^[^ ^149^ ^]^ the treatment of bare GO or rGO may induce MMP changes due to internalization and contact with the inside cells of mitochondria. The mitigation of MMP changes is largely associated with the reduction of charge transfer between graphene (both negatively charged GO and positively charged p‐G) and mitochondria by coating the graphene materials with polymers or proteins and generating a neutral contact surface area.^[^ ^118^, ^141^ ^]^

##### Cell Starvation

A study showed that cell starvation occurs when proteins and other cell nutrients are attracted to the charged functional groups on the surface of graphene derivatives.^[^ ^16^ ^]^ However, a limited study was performed, and liver cell starvation is considered an example when treated with GO. GO in hepatocytes tries to remove high and low molecular weight serum components and nutrients and finally causes cell starvation.^[^ ^1^ ^]^ Therefore, it is supposed that modification methods that increase steric hindrance could be an option to solve this problem.

##### Cell Physical Damages

The two main causes of physical damage in cells include the direct damage of cell membranes caused by the sharp edges of graphene materials, thereby destroying cell structures,^[^ ^148^ ^]^ and the strong dispersion and hydrophobic interaction between the p‐G graphene and lipids, leading to the direct extraction of phospholipids out of the lipid bilayer.^[^ ^49a^ ^]^ GO was found to reduce reproductive ability by inducing physical damage during the gonad development of *Caenorhabditis elegans*.^[^ ^119^ ^]^ Research on the interaction between p‐G and brain microvascular endothelial cells demonstrated increased lactate dehydrogenase release at doses starting at 2.5 µg mL^−1^,^[^ ^150^ ^]^ indicating the extraction of phospholipids and cell necrosis. According to several groups, coating with biocompatible polymers or proteins like PEG^[^ ^151^ ^]^ and bovine serum albumin (BSA)^[^ ^49a^ ^]^ largely reduced physical damage.

##### Gene Toxicity

RNA sequencing was utilized based on the NovaSeq 6000 sequencing system for the pathway analysis and gene ontology enrichment analysis of the differentially expressed genes caused by GO exposure (Figure 5d).^[^ ^139^ ^]^ A significant difference between acute and long‐term exposure was noticed. Acute exposure of 8–80 µg mL^−1^ for 48 h resulted in mitochondrial dysfunction without apparent cell death. Long‐term exposure of 1–5 µg mL^−1^ for 28 days caused a gradual sensitization to apoptosis induction, upregulation of proapoptotic genes of the Bcl‐2 family and downregulation of antiapoptotic genes classified in the inhibitors of apoptosis (IAP) family. To pacify the up/downregulation of genes in lung cells, inhibitors such as AT‐101 and ABT‐199 targeting the Bcl‐2 family can also be utilized.

To summarize, cytotoxicity induced by bare graphene materials mainly involves five routes. The cytotoxicity could be reduced by controlling dose, size, thickness, functional groups, and functionalization methods. However, it is noticed that similar graphene materials induce different cytotoxicity during exposure to various cell lines; for example, with the same treatment, A549 cells obtained less cell viability than BEAS‐2B.^[^ ^46a^ ^]^ A study suggested that A549 cells exhibited more phagocytic activity and, thus, are more sensitive to graphene.^[^ ^46a^ ^]^ It is an emerging study investigating the basic mechanisms; two assumptions have been proposed. 1) From the synthesis process, even with the same synthesis and functionalization methods, sizes, and thickness would be in a range instead of an exact value. As sizes/thickness influence cell uptake, the difference in cell viability may be induced. This is the reason repeatability (reduced variance) is always emphasized. 2) From the point of cell lines, it is supposed that graphene may activate different sites of DNA or mRNA in different cells and produce various cytokines. Some cytokines may facilitate cell apoptosis, and some mitochondria in a few cells may produce ROS or change MMP when treated with graphene. Therefore, multifunctionalization can probably be a method to improve nanosafety when applied in vivo.

#### 4.1.2 In Vivo Biodistribution and Histology Changes

In vivo tests are mainly applied by intravenous administration, intraperitoneal injection, inhalation, and oral administration. Previous research has always utilized labeling methods to track the biodistribution of graphene materials in important tissues. As different from the cell viability assays used in in vitro tests, histology analysis is beneficial to directly evaluate the effects such as fibrous, proliferation, or oedema induced by exposure to graphene materials. Furthermore, the degradation of these nanomaterials is also important in evaluating nanosafety, where urinalysis is commonly used.

This section describes the biodistribution of different graphene materials, including accumulation and excretion routes, followed by the introduction of histology changes due to inflammatory or immune responses.

##### Biodistribution of Different Kinds of Graphene Materials

**Figure 6:**
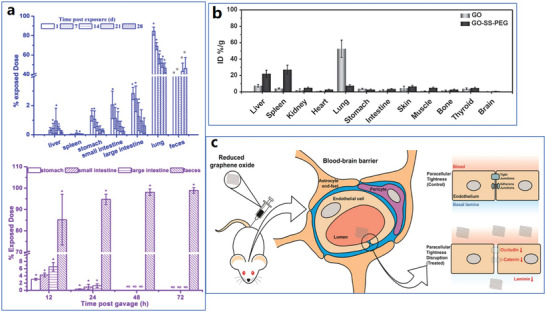
Biodistribution of different graphene materials. a) Biodistribution and excretion of FLG (5 µg) after intratracheal instillation and oral gavage at different times. Reproduced with permission.^[^ ^12a^ ^]^ Copyright 2016, Springer Nature. b) Biodistribution after intravenous administration of 4 mg kg^−1131^I‐labeled GO–SS–PEG (30 nm) and GO (400–500 nm) in Balb/c mice. Reproduced with permission.^[^ ^122^ ^]^ Copyright 2013, Wiley‐VCH. c) Schematic representation of the mechanism of time‐dependently permeabilized BBB induced by systemically injected rGO. Reproduced with permission.^[^ ^57b^ ^]^ Copyright 2015, Springer Nature.

To investigate the biodistribution of graphene materials, labeling methods, including fluorescence dyes,^[^ ^8^, ^152^ ^]^ radioactive labeling,^[^ ^12^, ^29^ ^]^ and enhanced imaging methods, including confocal mapping,^[^ ^151^ ^]^ matrix‐assisted laser desorption/ionization (MALDI), and mass spectrometry imaging (MSI)^[^ ^57b^ ^]^ were utilized. This section reviews the biodistribution of bare and functionalized graphene materials from p‐G, GO, and rGO. Carbon‐14 labeled FLG in mice after oral gavage or intratracheal instillation was tracked for 72 h and 28 days, respectively.^[^ ^12a^ ^]^ It was demonstrated that ^14^C‐FLG mainly accumulated in the large intestine and lungs by oral gavage or intratracheal instillation. After 14 days, 1% and 0.18% intratracheally instilled FLG were also detected in the liver and spleen, respectively, indicating that the FLG passed through the air‐blood barrier (**Figure** **6**a). For oral gavage, FLG was not absorbed into blood circulation through the gastrointestinal tract.^[^ ^12a^ ^]^ Moreover, after 28 days, 46.2% of the intratracheally instilled FLG was excreted through the feces as removed by mucociliary clearance and swallowed into the digestive system, while 47% was retained in the lungs (Figure 6a). Similar research on ^14^C‐FLG was carried out by Lu et al.^[^ ^30^ ^]^ Small‐FLG of 20–40 nm and large‐FLG of 330–630 nm were intravenously injected into mice. It was found that the amount of small‐FLG in the liver remained at ≈80%, even 180 days after administration, while a reduction of ≈70% of large‐FLG was recorded. The degradation process of large‐FLG was reported in their study. Large‐FLG first induced membrane perturbation of red blood cells, enhanced the erythrophagocytosis of Kupffer cells in the liver, caused degradation of hemoglobin into hemes and an increased iron concentration which facilitated the Fenton reaction to generate ·OH, and finally triggered the degradation of large‐FLG into ^14^CO_2_ for safer application. Sasidharan et al. studied acute and chronic toxicity of 20 mg kg^−1 99m^Tc labeled FLG, FLG‐COOH, and FLG‐PEG intravenously administered in mice for three months.^[^ ^29^ ^]^ The accumulation of FLG and FLG‐COOH was observed in the lung, liver, spleen, and kidney without uptake and retention in the brain, heart, and testis. The liver and spleen internalize FLG‐PEG without causing noticeable toxicity or organ damage. Moreover, based on the significant alteration of its Raman signature, it is supposed that FLG‐PEG could be cleared from reticuloendothelial systems (RES) after one month.

Zhang et al. examined the biodistribution and biocompatibility of ^188^Re‐GO in a mouse model based on the radiotracer technique after intravenous injection. High uptake and long‐term accumulation of GO were found in the lung, while the uptake by RES was low.^[^ ^48^ ^]^ GO accumulation in the lung was supposed to be because of the instability of uncoated GO in the physiological environments.^[^ ^153^ ^]^ At the same time, functionalized GO–SS–PEG accumulated in the liver and spleen, and these RES could clear graphene by macrophage uptake (Figure 6b).^[^ ^122^ ^]^ Yang's group also studied the in vivo behavior of ^125^I‐labeled GO–PEG in the mice model.^[^ ^154^ ^]^ With intravenous administration of 20 mg kg^−1^, the graphene mainly accumulated in RES, such as the liver and spleen, while negligible accumulation was found in the lung. Graphene was gradually cleared by renal and fecal excretion in three months without causing significant toxicity.^[^ ^155^ ^]^ It is also proposed that graphene with a size <10 nm could be cleared through renal excretion, and larger graphene could be excreted via the biliary pathway into faces.^[^ ^154^ ^]^

Mendonça et al. studied the distribution of rGO by MALDI and MSI (Figure 6c),^[^ ^57b^ ^]^ and rGO was mainly distributed in the thalamus and hippocampus among brain tissues. After treatment with rGO, the paracellular pathway was weakened, which further induced a time‐dependent permeabilized blood‐brain barrier (BBB). Syama et al. utilized confocal Raman mapping to detect the biodistribution and blood clearance of PEG–rGO on mice modules based on intraperitoneal and intravenous administration.^[^ ^151^ ^]^ It has been found that PEG–rGO could travel to the brain, liver, kidney, spleen, and bone marrow. PEG–rGO exposure to the liver, spleen, and kidney induced hepatotoxicity and immune responses in the first few days. The presence of PEG–rGO in the brain indicated that it could pass through the BBB. A small amount of PEG–rGO was also excreted via urine for clearance.

In conclusion, graphene biodistribution and graphene uptake by oral gavage cannot be absorbed into blood circulation through the gastrointestinal tract and is mainly cleared via feces in 72 h. Bare graphene that is intratracheally instilled or intraperitoneally/intravenously administrated is mainly accumulated in the lung, and the smaller the size, the harder it is to excrete. Functionalized graphene with smaller sizes is mainly aggregated in RES, and the excretion routes include macrophage uptake and renal and fecal excretion. Especially, rGO or functionalized rGO could pass through or interrupt the BBB and distributed into the brain.

##### Histological Changes due to Inflammatory or Immune Responses

The mechanisms of inflammatory or immune responses caused by naked and functionalized graphene materials are first introduced, followed by typical induced histological changes. In inflammatory response, some toxic graphene materials may activate immune cells to produce glycoproteins and cytokines, which then attract more immune cells triggering indiscriminate attack, thus killing both graphene‐internalized cells and normal cells and even inducing histology changes in normal tissues. According to previous research, inflammatory or immunoreactive responses were detected by FCM,^[^ ^111b^ ^]^ quantitative RT‐PCR (qRT‐PCR) analysis,^[^ ^29^ ^]^ or western blotting tests.^[^ ^57b^ ^]^

**Figure 7:**
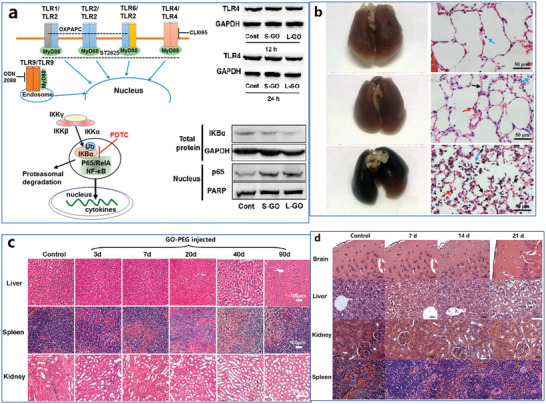
Immune/inflammatory response mechanism and histology changes induced by different graphene materials. a) Mechanism of M1 polarization induced by interaction with TLRs, mechanism of enhancing transcription based on NF‐*κ*B activation, and the corresponding results of the Western blotting analysis. Reproduced with permission.^[^ ^113^ ^]^ Copyright 2015, American Chemical Society. b) Lungs were harvested 24 h post intratracheal instillation of the control group, 5 and 50 µg FLG, where blue arrows indicate cells in alveoli, red arrows meant parenchymal. Black arrows demonstrate interstitial oedema. Reproduced with permission.^[^ ^12a^ ^]^ Copyright 2016, Springer Nature. c) H&E stained images of the control group's liver, spleen, and kidney and GO–PEG (20 mg kg^−1^) post intravenously injected at various times, where negligible organ damage or lesion was observed. Reproduced with permission.^[^ ^154^ ^]^ Copyright 2011, American Chemical Society. d) Histopathological examination of tissues from mice in the control group and post intraperitoneally injected PEG–rGO (20 mg kg^−1^) at different times. Reproduced with permission.^[^ ^151^ ^]^ Copyright 2017, Elsevier Ltd.

A typical immune cell macrophage was applied to interpret how graphene materials activate inflammatory or immune responses. It is known that inflammatory and immunogenic responses are caused by M1 polarization^[^ ^113^ ^]^ and transcriptions.^[^ ^143^ ^]^ Both naked p‐G, GO, and rGO may induce M1 polarization, while transcriptions are only caused by GO/rGO due to cell internalization. During M1 polarization of macrophages, graphene materials facilitated the secretion of proinflammatory cytokines such as Interleukin‐8 (IL‐8), IL‐1*β*, IL‐6, IL‐10, tumor necrosis factor (TNF), and IL‐12p70 in peripheral blood‐derived mononuclear cells. It was noticed that p‐G induced higher expression of IL‐8 and IL‐6 than GO.^[^ ^1^ ^]^ These cytokines then activated inflammatory and immunogenic responses. It was reported that larger GO was more likely to remain on the plasma membrane instead of penetrating cell, which induced more robust interactions with toll‐like receptors (TLR) and more activation of NF‐*κ*B pathways; thus, M1 polarization that enhanced proinflammatory cytokine production and engaged immune cells was promoted by larger GO (**Figure** **7**a).^[^ ^113^ ^]^ However, it is different when applying p‐G. Cicuéndez et al. found flavin mononucleotide‐stabilized pristine graphene (pG‐FMN) induced macrophage proinflammatory responses, especially in smaller sizes.^[^ ^156^ ^]^

M1 polarization was mainly caused by the accumulation of graphene on the cell membrane; thus, smaller GO/rGO and larger p‐G would reduce M1 polarization. When GO/rGO is internalized by macrophages via endocytosis, it may be delivered to nuclear by endosome and cause transcripts of proinflammatory genes. For example, several groups reported GO and rGO triggered NF‐*κ*B activation (Figure 7a).^[^ ^113^ ^]^ But unlike GO, rGO could not fully transcript proinflammatory genes due to the lack of additional transcription factors.^[^ ^143^ ^]^ Size control is difficult to solve as it only regulates cell uptake of graphene materials. Surface modifications that reduce the direct contact between graphene and DNA might be a solution. PEG–GO did not induce such activation and would not cause side effects, including enhanced nitric oxide production, increased intracellular succinate, itaconate, phosphocholine, and phosphocreatine.^[^ ^156^, ^157^ ^]^ Also, BSA^[^ ^122^ ^]^ and PVP^[^ ^141^ ^]^ coating could largely reduce the inflammatory response.

Apart from activating immune cells, immunosuppression is another area that needs consideration. Sasidharan et al. tested whether graphene provides cytotoxicity to immune cells.^[^ ^1^ ^]^ They observed no difference between PHA‐M alone treated cells and graphene and PHA‐M treated cells. Thus, both p‐G and f‐G did not affect impeding innate lymphocyte proliferation. In conclusion, suitably functionalized graphene materials neither stimulate nor suppress the regular function of immune cells.

Histological changes induced by graphene materials can present an intuitive evaluation of in vivo behaviors. Inhalation of naked p‐G induced large accumulation in the lung, with concentrations larger than 50 µg mL^−1^, and neutrophil infiltration was found, indicating an inflammatory response.^[^ ^12a^ ^]^ Moreover, this further led to lung oedema, where the lungs lose their ability to pump fluid out of the space (Figure 7b).^[^ ^12a^ ^]^ After intravenous administration of 200 µL, 0.1 mg mL^−1^ naked p‐G, accumulation in Kupffer cells in the liver was found, and no significant inflammatory changes were induced.^[^ ^30^ ^]^ However, another group utilized 20 mg kg^−1^ FLG and FLG‐COOH. Aggregation was found in the liver and spleen, leading to severe cellular damage, including acute to chronic inflammation, pulmonary oedema, granuloma formation, interstitial nephritis, and structural damage without appreciable clearance.^[^ ^29^ ^]^ The p‐G dose influenced toxicity. More activation to macrophages with a larger dose, thus caused more inflammatory and immune responses and finally induced histological changes. FLG‐PEG was mainly internalized by the liver and spleen without causing noticeable toxicity or organ damage.^[^ ^29^ ^]^

For naked GO, with a dose larger than 20 mg kg^−1^, GO elicited severe pathological changes of granuloma formation in the lung, liver, spleen, and kidney.^[^ ^149^, ^158^ ^]^ In comparison, 10 mg kg^−1^ GO did not cause significant pathological changes to organs.^[^ ^48^ ^]^ Also, functionalization significantly reduced the toxicity; for instance, 20 mg kg^−1^ PEG–GO induced negligible damage in three months (Figure 7c).^[^ ^154^ ^]^ The size of naked GO was also found to influence the toxicity to the lung; GO with the size of 50–300 nm was demonstrated to induce less damage. Single to few‐layer GO sheets were applied by Rodrigues et al., where both the acute (1 and 7 days) and chronic (28 and 90 d) pulmonary responses were evaluated after single intranasal instillation (50 µg) in mice.^[^ ^159^ ^]^ Larger GO (lGO, 1–30 µm), small GO (sGO, 50 nm to 2 µm), and ultrasmall GO (usGO, <300 nm) were synthesized via modified Hummer's method. According to their results, larger lateral size induced stronger pulmonary inflammation in acute response and more significant pulmonary infiltration and granulomatous response in 28 days, while the response recovered in 90 days. The translocation to the lung was found to be in the order: lGO < usGO < sGO at 1 day, while the deposition of graphene materials in the lower respiratory tract was the opposite, and these graphene materials obtained different translocation routes due to size differences with time varied. The time‐dependent gene expression patterns were demonstrated via RNA sequencing of these three sized GO, and a hierarchical cluster was applied for analysis, IFN‐𝛾 and TNF‐*α* were the main regulators of the affected pathways by l‐GO. The long‐term study revealed that though lGO induced granuloma formation and persisted till 90 days, no pulmonary fibrosis was observed, attributed to the biotransformation of GO into less graphitic structures (less reactive) according to Raman imaging.

For naked rGO, Mendonça et al. found transient BBB breakdown based on systemic injection.^[^ ^57b^ ^]^ Nevertheless, this disruptive effect on the BBB was transient and reversible. For functionalized rGO, Syama et al. reported exposure of PEG–rGO to the liver, spleen, and kidney‐induced hepatotoxicity and immune responses in the first few days. However, these side effects soon recovered with no obvious toxic clinical signs after 14 days without repeated administration (Figure 7d).^[^ ^151^ ^]^

Therefore, with reasonable control of dose, sizes, administration pathways, and functionalization methods, the inflammatory or immune responses can be reduced, and less histological damage would be induced in vivo.

**Figure 8:**
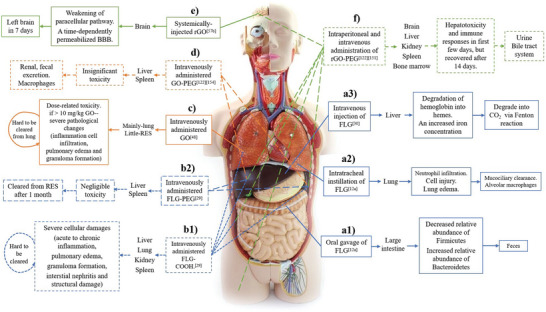
In vivo biodistribution, histological changes, and clearance routes of typical graphene materials. a1,a2) Routes apply FLG (60 to 590 nm, 0.25 mg kg^−1^).^[^ ^12a^ ^]^ a3) Route utilizes FLG (1 mg kg^−1^) with both small (20 to 40 nm) and large sizes (330 to 630 nm), where large‐FLG can be degraded.^[^ ^30^ ^]^ b1,b2) Pathways correspond to the functionalized FLG (20 mg kg^−1^) of FLG‐COOH (128 ± 37.6 nm) and FLG‐PEG (115 ± 28.4 nm).^[^ ^29^ ^]^ c) Route uses GO with different doses (10–800 nm,1 mg kg^−1^, and 10 mg kg^−1^).^[^ ^48^ ^]^ d) Route shows behavior and effects after treatment of GO–PEG with different sizes and doses (30–50 nm, 4 mg kg^−1^;^[^ ^122^ ^]^ 10–30 nm, 20 mg kg^−1[^ ^154^ ^]^). e) Pathway demonstrates the interaction between rGO (342 ± 23.5 nm, 7 mg kg^−1^) and the brain.^[^ ^57b^ ^]^ f) Route administrates rGO–PEG with different sizes (1000 nm,^[^ ^151^ ^]^ 50 nm,^[^ ^122^ ^]^ 10 mg kg^−1^).

The in vivo biodistribution, histological changes, and clearance routes of typical graphene materials based on mice models were summarized in **Figure** **8**. Since animal experiments can predict the situation in humans to some extent, thus, a human body model was applied for better understanding (Figure 8).^[^ ^160^ ^]^ For better comparison, the units of doses were unified as mg kg^−1^, where the weight of the tested mice was generally 20 ± 2 g based on the utilized mice in previous research.^[^ ^126^, ^137^ ^]‐^

#### 4.1.3 Mitigation of Toxicity

In summary, the main routes for graphene materials to induce toxicity to cells include ROS generation, MMP depletion, cell starvation, physical damage, gene toxicity, and triggering inflammatory or immune responses in vivo. In order to pacify the cytotoxicity, functionalization methods that provide graphene materials with a biocompatible and neutral charged surface, as well as steric hindrance, are necessary, where multifunctional is highly recommended. In terms of mitigating toxicity in vivo, researchers should be concerned not only with the particular effects of graphene on specific tissues but the systematic distribution, series cascade action (like graphene‐activated macrophages, then immune cells attack normal tissues) and excretion routes. It is proposed that reasonable control of dose (<20 mg kg^−1^), sizes (50–1000 nm), and functionalization methods (functionalized with polymers, biomolecules, or proteins) could reduce the inflammatory or immune responses, induce less histological damage, and improve the excretion from the body.^[^ ^126^, ^161^ ^]^

### 4.2 Biocompatibility

Biocompatibility is the ability of nanomaterials to interact with cells, tissues, or bodies without causing damage, and hemocompatibility is generally employed to evaluate the biocompatibility of nanomaterials.^[^ ^162^ ^]^ This section introduces studies on the influence of nematode growth and heredity behavior, hemolytic potential, platelet activation, platelet aggregation, and plasma coagulation after graphene exposure to human peripheral blood.

Nematodes, particularly *C. elegans*, are used widely in biomedical tests due to their properties, including short life cycle, high reproductive rate, ease of feeding, etc.^[^ ^163^ ^]^ Bare graphene materials were found to be harmful to *C. elegans*. Both graphene and GO induced behavioral deficits and neurotoxicity in *C. elegans* but GO was more toxic than graphene.^[^ ^150^ ^]^ GO induced long‐term side effects, where reproductive ability was reduced due to the damage of the gonad of *C. elegans* during development.^[^ ^119^ ^]^ Functionalized graphene materials would induce an ignorable negative effect on biocompatibility, and the side effects induced by bare graphene were related to the RNA expression. Rive et al. provided a more comprehensive study from the effect of acute (12 h, 1, 10, 100, and 200 µg mL^−1^) and chronic (72 h, 100 and 200 µg mL^−1^) GO and GO–NH_2_ treatment in *C. elegans*, either acute or chronic GO treatment induced more negative effects than beneficial effects. By contrast, GO–NH_2_ treatment did not cause any detrimental effects.^[^ ^163a^ ^]^ Chronic GO exposure induced a decreased size of *C. elegans* and abnormity in morphology, while acute treatment increased the mobility of *C. elegans*, which was similar to the exposure to pathogenic bacteria, indicating an aversion response. RNA sequencing data demonstrated immunotoxicity induced by GO, where at least 55 genes that act in innate immunity to bacteria were overrepresented when exposed to GO. Additionally, the gene expression by *C. elegans* with acute GO and pathogenic PA14 bacteria exposure was mildly correlated, though it was supposed that the pre‐exposure of GO could immunize *C. elegans* to tolerate lethal pathogen exposure better; the adverse detrimental effects may outweigh the beneficial effects. Like previous research, functionalized GO–NH_2_ was found to moderate activating the innate immune response, increase cellular stress tolerance, and extend lifespan through the hormesis process while inducing ignorable side effects. However, as a short life‐span animal, *C. elegans* could provide limited information on long‐term exposure effectiveness and safety of GO–NH_2_ if applied in mammal animals.

Hemolysis is induced by the loss of membrane integrity of RBCs. It causes leakage of hemoglobin (Hb) into the blood plasma,^[^ ^123^ ^]^ and generally, hemolysis of less than 1% was required for optimal biomedicine.^[^ ^120^ ^]^ It is supposed that graphene may obtain hemolytic potential, and the hemolytic property is related to the size, thickness, surface charge, chemical composition, and concentration of graphene materials.^[^ ^120^ ^]^ Intravenous injection of large‐FLG into mice can cause hemolysis of RBCs.^[^ ^30^ ^]^ Bare GO and rGO damage the integrity of RBCs by electrostatic interactions,^[^ ^151^ ^]^ while functionalization that neutralized the surface charge induced insignificant hemolysis.^[^ ^123^ ^]^ Treating RBCs derived from human peripheral blood with p‐G or f‐G did not show significant hemolysis up to 75 µg mL^−1^; thus, it is important to pay attention to the concentration applied.^[^ ^1^ ^]^ Activated blood platelets would release platelet factor 4 (PF4), inducing blood clotting and platelet aggregation and finally causing thrombogenesis.^[^ ^123^ ^]^ Therefore, studies on whether graphene induces platelet activation, aggregation, and thromboembolism are necessary. Previous research reported that GO is more likely to induce thrombus and platelet activation than rGO.^[^ ^1^ ^]^ Singh's group proposed GO elicited aggregation response in platelets by activating nonreceptor protein tyrosine kinases of the Src family and releasing intracellular free calcium from cytosolic stores.^[^ ^87^ ^]^

Moreover, rGO does not induce platelet activation due to reduced charge density on the rGO surface. At the same time, the prothrombotic character of GO was dependent on the distribution of surface charge.^[^ ^87^ ^]^ Besides using rGO, functionalization methods like dextran functionalized graphene oxide (GO‐Dex) were also demonstrated to avoid platelet activation.^[^ ^123^ ^]^

The basic principle of plasma coagulation is activating factor X (a serine endopeptidase) to Xa, which activates prothrombin to thrombin and converts fibrinogen to fibrin.^[^ ^87^ ^]^ Generally, plasma coagulation can be divided into intrinsic and extrinsic pathways, which correspond to prothrombin time (PT) and activated partial thromboplastin time (aPTT) factors, respectively.^[^ ^1^ ^]^ Based on the results that both PT and aPTT coagulation time factors of graphene‐treated samples were in a normal range, it was confirmed that graphene would not interfere normal function of platelets nor influence coagulation pathways.^[^ ^120^ ^]^

In summary, bare graphene materials would induce hemolysis and platelet activation in a dose/size‐dependent manner. Functionalization methods could further decrease graphene damage to RBCs and reduce platelet activation induced by GO.

### 4.3 Status and Challenges for Commercial Biomedical Application

New drugs and biological products for people must be FDA/ICH/EMA approved before being marketed commercially, depending on the region. The approvements are comprehensive and strict, generally evaluated from four aspects,^[^ ^164^ ^]^ including quality control in the synthesis process (stability, analytical validation, impurities identification, specifications demonstration, quality risk management, etc.), safety evaluation in animal experiments (carcinogenicity, genotoxicity, toxicokinetic and pharmacokinetic studies, toxicity testing, reproductive toxicology, etc.), efficiency analysis in clinical research (clinical safety for drugs used in long‐term treatment, pharmacovigilance, dose‐response studies, factors include ethnic and age, etc.), and regulations for multidisciplinary (medical terminology (MedDRA), the Common Technical Document, and the development of Electronic Standards for the Transfer of Regulatory Information, etc.). Generally, good manufacturing practice (GMP) certification portends a higher probability of approval from FDA/ICH/EMA, as passing GMP regulations reflects that production is under proper design, monitoring, and quality control. By contrast, product safety, quality, and functionality can be ensured.^[^ ^165^ ^]^ Though GMP regulations in different regions have different principles and contents,^[^ ^166^ ^]^ comprehensive evaluation of the cytotoxicity, sensitization, irritation, acute toxicity, material pyrogenicity, subchronic toxicity, genotoxicity, implantation, hemocompatibility, chronic toxicity, carcinogenicity, neurotoxicity, and degradation of graphene materials are recommended if aiming at commercial biomedical applications.

A long period is required before obtaining approval; FDA approval, for example, takes an average of 12 and 7 years for drugs and devices, respectively. The administration process is especially challenging for new drugs or devices based on a new concept; for instance, the concept of gene therapy was shown as early as 1972, while the approval of that for commercial application was obtained in 2003.^[^ ^167^ ^]^ As graphene materials were discovered and developed in 2004, the evaluation of toxicity, biocompatibility, biodegradation, and treatment efficiency had not comprehensively proceeded. It was no wonder that graphene materials for commercial biomedical applications with minimal approval from FDA/ICH/EMA. Commercial biomedical application of graphene is recommended to be started from Class I and II medical devices with minimal risks and minimally invasive to the human body. A hand‐held IPL device (JOVS Graphene Hair Removal Device) approved by FDA in 2021 as a Class II medical device would be a good instance where the safety and effectiveness are easier to evaluate with those in vivo products. There have been negative comments on graphene materials due to the unguaranteed quality of raw materials and few successful application cases; the development and commercial application of those devices under strict and comprehensive approval is expected to win the reputation and expand market share. Apart from the medical device, a graphene‐based magnetic resonance imaging (MRI) contrast agent was reported to be prepared for FDA approval. The dextran functionalized water‐soluble Mn^2+^ intercalated graphene assessed eight key in vitro physicochemical properties required by FDA.^[^ ^168^ ^]^ Evaluation from osmolality, viscosity, partition coefficient, protein binding, thermostability, histamine release, relaxivity, and in vitro phantom MRI instead of just imaging quality is indeed more comprehensive than most research and development in MRI contrast agents.

Challenges for commercial biomedical application of graphene materials are mainly attributed to the unguaranteed quality of raw graphene materials, insufficient study of large‐scale, stable, and low‐cost production, long‐term clinical safety, and treatment efficiency of graphene material targeting specific applications. Malhotra et al. comprehensively studied the cytotoxicity of 36 commercial graphene materials. The physical and chemical properties of these graphene materials varied greatly, while contaminates contained in nearly all these materials played the most important role in inducing toxicity.^[^ ^169^ ^]^ According to their results, the 50th percentile (N^50^) of the graphene with layer <10, while none of the samples with the 90th percentile of layers <10, and the size ranges from 0.5 to 1.5 µm among the samples. *I*_D_/*I*_G_ was defined as 1.3 as a threshold for intact and defective graphene, where 23 samples with values >1.3. Additionally, the wt% of carbon atoms exhibited a wide distribution among the samples. Impurities in samples were demonstrated from XPS results; intact graphene showed nine out of the ten lowest sp^2^ ratios among the samples, indicating the presence of surface contaminants, including adventitious carbon, solvent, or surfactant residues. Based on the cell viability and cytotoxicity tests, it was proposed that the toxicity was determined by multiple parameters, including size, shape, contaminates, and structures. By contrast, the contaminants were the most important factor. Though removing the contaminates could reduce toxicity, and the problem must be handled from the source with two means. First, it was found that safety datasheets provided by graphene suppliers were either unavailable or inadequate. If comprehensive and strict standards on safety and toxicity evaluation from a regulatory agency are imposed, the development and application of commercial graphene materials will obtain a greater promotion. Second, the existing large‐scale generation methods such as ECAME,^[^ ^24^ ^]^ shear mixing,^[^ ^26^, ^33^ ^]^ FJH,^[^ ^64^ ^]^ and arc discharge^[^ ^61^ ^]^ are reported to produce raw graphene materials either with high‐quality or low‐cost; however, methods that achieve producing high‐quality, uniform sizes graphene with low‐cost and environmental friendly still need further study. Potential solutions include low‐cost biomass as a precursor,^[^ ^170^ ^]^ combining synthesis methods, and reducing equipment costs. Challenges in evaluating long‐term clinical safety and treatment efficiency of graphene would be easier with high‐quality and standard raw materials; also, it is recommended to carry on the evaluation based on the GMP regulations introduced in the last paragraph and refer to FDA/ICH/EMA approval policy. It is noteworthy that different criterion is applied when facing specific application directions; for instance, the graphene‐based wound dressing requires completely different toxicity and efficiency criteria compared with graphene working as a drug carrier. Furthermore, for researchers to do cytotoxicity or biocompatibility evaluation based on graphene materials, it needs to be noticed that cell lines influence the cell viability results. Choosing cell lines based on the ISO guidelines or reputational literature is recommended.

## 5 Biomedical Applications

**Figure 9:**
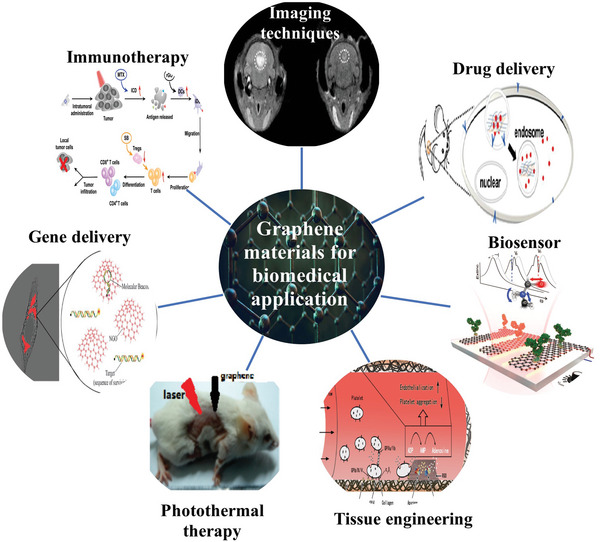
Illustration of the graphene materials research for biomedical application. Imaging technique image: Reproduced with permission.^[^ ^6^ ^]^ Copyright 2017, Elsevier Inc. Drug delivery: Reproduced with permission^[^ ^173^ ^]^ Copyright 2014, American Chemical Society. Biosensor: Reproduced with permission.^[^ ^174^ ^]^ Copyright 2015, The American Association for the Advancement of Science. Tissue engineering: Reproduced with permission.^[^ ^175^ ^]^ Copyright 2017, American Chemical Society. Photothermal therapy: Reproduced with permission.^[^ ^2^ ^]^ Copyright 2010, American Chemical Society. Gene delivery: Reproduced with permission.^[^ ^176^ ^]^ Copyright 2010, Royal Society of Chemistry. Immunotherapy: Reproduced with permission.^[^ ^11^ ^]^ Copyright 2020, Elsevier Ltd.

With a clear understanding of the toxicity and biocompatibility of graphene materials, many research groups highlight the potential of using graphene materials in biomedical fields. Apart from 2D graphene materials, 1D carbon nanotubes (CNTs) also attracted researchers in biomedical applications. However, CNT was less effective in some functionalization and biomedical applications and induced more toxicity than graphene. In a study of BSA‐functionalized carbon materials, the adsorption capacity of BSA by GO was eight times that of single wall CNT, attributed to the smaller surface area of CNT.^[^ ^171^ ^]^ rGO–PEG was demonstrated to be a low‐cost NIR photothermal agent compared with Au nanoparticles and CNT, as rGO obtained a lower mass extinction coefficient.^[^ ^76^ ^]^ In terms of in vivo toxicity, single wall CNT accumulated in the spleen and liver, while it was not observed with GO–PEG; size and shape of the carbon material may be the main factors.^[^ ^162^ ^]^ Though CNT illustrates advantages in several fields, complementary DNA was reported to be more stable during attachment on CNT compared with graphene.^[^ ^162^ ^]^ As the synthesis, functionalization, toxicity, and biomedical application of CNT are outside the specific scope of this article, readers interested are advised to check the related review papers.^[^ ^172^ ^]^ A classification of biomedical applications based on graphene materials into seven parts, including imaging techniques, drug delivery, biosensor, tissue engineering, photothermal therapy, gene delivery, and immunotherapy, is shown in **Figure** **9**.

### 5.1 Imaging Techniques

Improving biomedical imaging techniques aims to provide higher resolution, better penetration depth, less damage to tissues, faster imaging speeds, higher signal‐to‐noise ratio, and multifunctional (structural and functional) modes. Cell internalization of graphene materials makes them possible to enhance cellular fluorescence imaging.^[^ ^98^, ^115^ ^]^ Graphene, with a large surface area to carry metal ions, targeting molecules, and labeling markers, is a potential material to enhance MRI, positron emission tomography (PET), and computed tomography (CT) imaging.^[^ ^21^ ^]^ Good laser absorption and vibration properties of graphene promoted the image qualities of photoacoustic imaging (PAI).^[^ ^25^ ^]^ This section describes the application of graphene materials in fluorescence imaging, MRI, PET, CT, and PAI.

#### 5.1.1 Fluorescence Imaging

As indicated in Sections 4 and 5, functionalized graphene materials attached with fluorescence molecules can be internalized by cells while inducing negligible toxicity. Thus, they are beneficial for improving cellular fluorescence imaging, especially for regions of interest (ROI) that are hard to detect, such as the brain. However, graphene materials quenched fluorescence (FL) dyes through the fluorescence resonance energy transfer (FRET) effect, which affected the signal of FL imaging.^[^ ^152^ ^]^ Hence, several methods have been developed to avoid the quench and improve FL imaging costs and efficiency.

**Figure 10:**
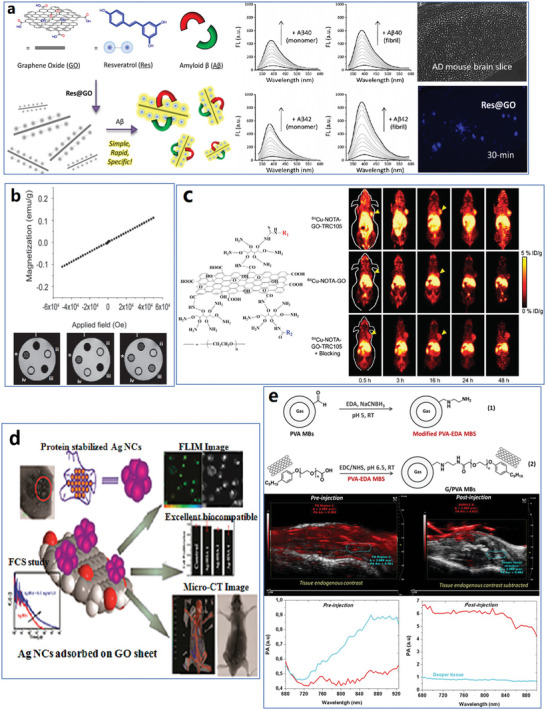
Functionalized graphene materials for improving five types of imaging techniques. a) Left: construction of Res‐GO for the rapid fluorogenic probing of A*β*; middle: FL titration spectra of Res‐GO (20 µm of resveratrol with 50 µg mL^−1^ GO) with increasing concentration of A*β* at 310 nm excitation; right: a bright‐field image of a mouse brain section with amyloid plaques and FL images of Res‐GO. Reproduced with permission.^[^ ^152^ ^]^ Copyright 2014, American Chemical Society. b) SQUID magnetization curve of FGO and T_2_ relaxation measurements with a 4.7 T MRI for FGO (* means control group, i, iii sites in the three graphs correspond to FGO with decreasing concentrations from left to right, while ii, iv sites correspond to GO with decreasing concentrations from left to right). Reproduced with permission.^[^ ^177^ ^]^ Copyright 2013, Wiley‐VCH. c) Schematic representation of ^64^Cu‐NOTA‐PEG‐GO‐TRC105 (R1 represents NOTA, R2 means TRC105) and serial coronal PET images of 4T1 tumor‐bearing mice at different time points postinjection of ^64^Cu‐NOTA‐GO‐TRC105. Reproduced with permission.^[^ ^102a^ ^]^ Copyright 2012, American Chemical Society. d) Synthesis, functionalization, and application of biocompatible Ag‐NCs/GO as contrast agent for enhancing CT imaging of bone tissues, where fluorescence lifetime imaging microscopy (FLIM) and fluorescence correlation spectroscopy (FCS) evaluated the internalization of the composite. Reproduced with permission.^[^ ^178^ ^]^ Copyright 2017, American Chemical Society. e) Synthesis and functionalization strategy of p‐G/PVA MBs, and real‐time in vivo PA images and PA signal spectra of subcutaneous injection of p‐G/PVA MBs into normal mouse hind limb. Reproduced with permission.^[^ ^25^ ^]^ Copyright 2016, American Chemical Society.

The real‐time detection of Amyloid *β* (A*β*) based on resveratrol (Res)‐GO with the advantages of cost and efficiency have been studied (**Figure** **10**a).^[^ ^152^ ^]^ A*β* is produced from the *β*‐amyloid precursor protein that is truncated successively by *β* and *γ* secrets, and it is associated with the pathology of AD. Res is a natural product widely found in red wine, which shows neuroprotective effects and binds specifically with A*β*. Res‐GO sensitively detected both A*β* monomers and fibers in a physiological buffer solution in 3 min, and fluorescent images of amyloid deposits in a mouse brain section could be derived within 30 min (Figure 10a). Pang's group demonstrated that PEG–GO conjugated with fluorophores could be utilized for intracellular imaging,^[^ ^115^ ^]^ and the GO‐mediated quenching of fluorescein luminescence was mitigated with PEG. PEG–GO could be taken up by the treated cells to present fluorescence imaging through confocal microscopy. The covalent binding of fluorescein on graphene through the polymer chain PEG 2000 could change the fluorescence,^[^ ^115^ ^]^ but the reason for PEG functionalization that avoids quenching needs to be further investigated. Quenching is supposed to mainly occur in noncovalent bonding, as the basic principle is FRET, which depends on the electrons’ movement. Electrons in the molecules that combine noncovalently are active and easy to transfer, while electrons in the covalently bonded molecules are difficult to transfer. Liu et al. utilized gelatine as a reducing reagent to synthesize gelatine–rGO and attached it with R6G for FL imaging.^[^ ^98^ ^]^ They proposed that glycans, proteins, and lipids in the cells could facilitate the recovery of fluorescence of R6G–gelatin–rGO.

#### 5.1.2 MRI

Graphene materials can be utilized in MRI as contrast agents to improve the relaxation efficiency and contrast and decrease the magnetic fields that need to be applied. Zhang's group produced PEG–GO first, then conjugated with a chelating agent 1,4,7,10‐Tetraazacyclododecane‐1,4,7,10‐tetraacetic acid (DOTA) and carried out chelation reaction with gadolinium (III). Finally, GO–DOTA–Gd complexes were synthesized and applied as a T_1_ MRI contrast agent.^[^ ^6^ ^]^ Their results show that the GO–DOTA–Gd complexes significantly increased the T_1_ relaxivity with an r_1_ value of 14.2 mm ^−1^s^−1^ at 11.7 T, three times higher than the commercially used Magnevist. They further treated human mesenchymal stem cells (hMSCs) with GO–DOTA–Gd complexes. Significant internalization and enhanced imaging intensity were observed without affecting the cell viability, growth, and differentiation behavior. A successful in vivo test of 5 × 10^5^ hMSCs labeled with GO–DOTA–Gd complexes also confirmed the potential application of this type of contrast agent. Fluorinated graphene oxide (FGO) without magnetic nanoparticles was reported to be another potential contrast agent for MRI imaging.^[^ ^177^ ^]^ Due to C—F bonds, FGO demonstrated paramagnetic behavior (Figure 10b), and FGO exhibited high SNR because of its scarce distribution of ^19^F compared with ^1^H in the human body. In vivo tests of such a graphene material were not reported in this work; perhaps toxicity and biocompatibility of FGO are required to be evaluated, and suitable functionalization methods need to be conducted in further studies.

#### 5.1.3 PET

The radiation markers used in traditional PET do not consider radiation safety and image quality and cannot specifically bind with tumor areas. Graphene materials that are radiolabeled and attached with ligands would be a good contrast agent. PEG–GO was conjugated to NOTA (for ^66^Ga‐labeling) and TRC105 antibody (binds to CD105, an ideal marker for tumor angiogenesis).^[^ ^102a^ ^] 66^Ga‐NOTA‐GO‐TRC105 synthesized could quantitatively evaluate the pharmacokinetics and tumor targeting efficacy based on PET imaging. In vitro tests demonstrated that the composites obtained excellent stability in complete mouse serum and were specific for CD105 in cell culture. In vivo tests with 4T1 tumor‐bearing mice showed that the composites were vasculature specific with little extravasation. They accumulated quickly in tumors and remained stable over time, and finally, the composites were cleared through the hepatobiliary pathway. Hong's group reported a similar outcome based on ^64^Cu‐NOTA‐GO‐TRC105 (Figure 10c).^[^ ^102a^ ^]^

#### 5.1.4 CT

CT is a well‐developed imaging technique, while recent research is concerned with using contrast agents that improve imaging quality and coordinate with PTT. Sun et al. synthesized PSS to functionalized GO‐gold nanorod (GNR) composites based on in situ growth method at room temperature.^[^ ^102b^ ^]^ To suit growth, aggregation of GNR should be avoided before attaching it to GO. According to their results, with an 808 nm laser (0.4 W cm^−2^) exposure for 6 min, the composites (50 µg mL^−1^) increased temperature from 25 to 49.9 °C. After connecting with CT agent iohexol, GO‐GNR also enhanced CT imaging. Thus, GO‐GNR could be utilized in precise CT‐image‐guided tumor PTT. In another work applied metal–graphene composite, human serum albumin (HSA) and BSA were applied first to mediate Ag nanoclusters (NCs), then the Ag‐NCs were noncovalently functionalization on the surface of GO.^[^ ^178^ ^]^ The composite was found to be internalized into the K562 cell (human erythroleukemic cell line) and enhance the contrast of bone tissues performed on mice models CT‐imaging. According to their results, 10 mm of the composite as a contrast agent increased the bone tissue volume in the imaging from 600–700 mm^3^ (control group) to 900–1000 mm^3^ (Figure 10d). Tough GO–metal composite is recommended to be applied when targeting on CT imaging enhancement, the dose of metal cluster concentration need to be carefully evaluated.

#### 5.1.5 PAI

PAI is still developed and examined in the laboratory as a novel imaging technique. Both invasive and noninvasive PAI can utilize graphene materials as contrast agents to improve imaging depth and quality since graphene materials exhibit good laser absorption and PA signal conversion abilities. Toumia et al. connected pristine graphene sheets with poly(vinyl alcohol) based microbubbles (PVA MBs) for the application of a contrast agent in photoacoustic imaging.^[^ ^25^ ^]^ In vivo test with an NIR laser, the composites (3.6 ± 0.8 µm diameter) greatly enhanced the PA signal of ROI without overlapping with signals of PA active endogenous molecules such as hemoglobin (Figure 10e). The biocompatibility and stability of p‐G/PVA MBs (30 µL, 3 × 10^7^ MBs mL^−1^, graphene content 5% w/w) were good during one week in the mice model.

### 5.2 Drug Delivery

Drug delivery based on functionalized graphene materials aims to send drugs to nidus while inducing less damage to normal tissues,^[^ ^38b^ ^]^ where stimuli responses can be applied to control drug release.^[^ ^22^ ^]^ Graphene materials possess a large surface area, supporting to carry of larger drug molecules. Functionalization methods reduce their toxicity and allow them to attach to both targeting ligands and specific drugs. In this part, the discussion of drug delivery is divided into basic targeting and advanced stimuli response release.

#### 5.2.1 Basic Targeting

Basic targeting allows accurate binding between the drug‐loaded graphene materials and the nidus. Drug molecules are generally attached to graphene via covalent and noncovalent bonding.^[^ ^179^ ^]^ Investigations have also explored the advanced graphene quantum dots (GQDs) fabricated from materials such as Clitoria ternatea^[^ ^72^ ^]^ (for the treatment of AD) that exhibit therapeutic effects. To target at nidus, ligands conjugation methods, including protein–glycoprotein receptor,^[^ ^130^, ^180^ ^]^ aptamers‐DNA/mRNA,^[^ ^181^ ^]^ and antibody–antigen^[^ ^142^ ^]^ are usually utilized, and these ligands bond to graphene via covalent methods. Especially, enhanced permeability is a passive targeting pathway to tumor cells,^[^ ^162^ ^]^ as the efficiency is not competitive.

Different functionalization methods provide graphene materials with different drug‐loading capacities. Kakran et al. covalently functionalized GO with Pluronic F38 (F38), Tween 80 (T80), and MD to support the delivery of the hydrophobic anticancer drug ellagic acid (EA).^[^ ^7^ ^]^ Among all these three functionalization methods, T80‐conjugated GO showed maximum loading capacity (1.22 g g^−1^) of EA due to less long hydrophobic chains and a larger surface area of GO. According to their results, the cytotoxicity of EA–GO was higher than free EA with in vitro tests of MCF7 and HT29 cells. At the same time, functionalized GO (<400 mg L^−1^) without EA did not cause significant cytotoxicity. Furthermore, the DPPH assay demonstrated that GO did not impede the antioxidant activity of EA.

Moreover, drug release efficiency in basic targeting methods is related to the molecular weights of composites. Gao et al. studied sustained antibiotic (levofloxacin) delivery by polysebacic anhydride (PSA) modification of GO based on esterification.^[^ ^182^ ^]^ The 2% GO‐modified PSA composites obtained nearly linear release behavior and an extended drug release for up to 80 days, with ≈95%. However, if GO absorbs water, an obvious reduction in the molecular weight of PSA was observed, which influenced the release behavior. In terms of GQDs synthesized directly from therapeutic materials for treatment, Kajal et al. fabricated GQD from Clitoria ternatea with microwave heating, aiming at treating Alzheimer's disease.^[^ ^72^ ^]^ The ctGQD increased inhibition of the acetylcholinesterase enzyme compared with donepezil; a higher level of glutathione and protein and decreased lipid peroxide and nitric oxide levels were observed.

**Figure 11:**
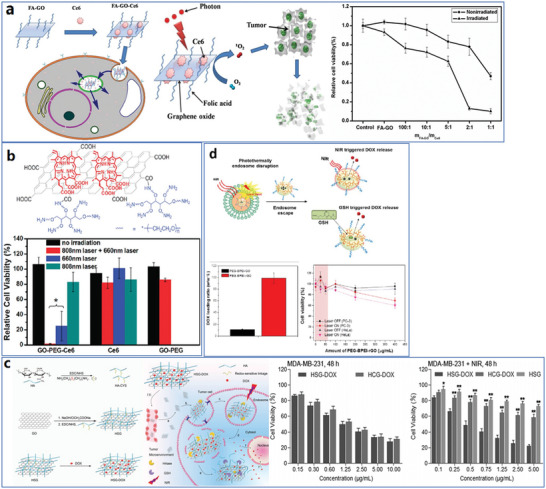
Functionalized graphene materials for drug delivery with different strategies. a) Mechanism of a basic targeting method using protein–glycoprotein receptor, where internalization of FA‐Ce6‐GO (0.5 mg mL^−1^) and the apoptosis process of tumors based on PDT is demonstrated. Reproduced with permission.^[^ ^180^ ^]^ Copyright 2011, Ivyspring International Publisher. b) GO–PEG–Ce6 was synthesized for photothermal enhanced PDT based on NIR irradiation with different wavelengths. Reproduced with permission.^[^ ^184^ ^]^ Copyright 2011, American Chemical Society. c) Synthesis of HSG‐Dox, and the process and results of stimuli‐responsive drug release based on NIR and redox. Reproduced with permission.^[^ ^130^ ^]^ Copyright 2017, Wiley‐VCH. d) PEG–BPEI–rGO conjugated doxorubicin (Dox) for NIR triggered endosome escape and GSH induced drug release, where the rGO obtained higher DOX loading ratio than GO, and the cell viability was found to decrease 20% with NIR irradiation. Reproduced with permission.^[^ ^185^ ^]^ Copyright 2013, American Chemical Society.

Regarding targeting methods, Huang's group used GO to deliver PS molecules for photodynamic therapy based on protein targeting.^[^ ^180^ ^]^ PSs are generally light‐sensitive porphyrin‐based molecules; when activated with light, PSs facilitate ROS generation and induce cell death (**Figure** **11**a). However, a previous study demonstrated that PS drugs accumulated nonspecifically due to their lipophilic or hydrophobic nature. In their research, folic acid (FA)‐conjugated chlorin e6 (Ce6) was loaded onto GO to support specific targeting and photodynamic‐induced apoptosis on MGC803 cells. For aptamer targeting, Tang's group functionalized GO with Cy5.5‐AS1411 aptamer and GO–Apt were used to enwrap Dox‐loaded mesoporous silica nanoparticles.^[^ ^181^ ^]^ Moreover, Yang et al. conjugated PEG–GO–NOTA–DOX to a monoclonal antibody (mAb) against Follicle‐stimulating hormone receptor (FSHR), making it possible for specific drug delivery.^[^ ^142^ ^]^

#### 5.2.2 Advanced Stimuli‐Responsive Release

Stimuli‐responsive methods based on intrinsic and extrinsic pathways are applied to achieve controlled drug release. Intrinsic routes involve stimulation of pH,^[^ ^109^ ^]^ redox,^[^ ^130^ ^]^ and biomolecules.^[^ ^142^ ^]^ Moreover, extrinsic methods include using NIR,^[^ ^181^, ^183^ ^]^ magnetic fields,^[^ ^109^ ^]^ and temperature variation.^[^ ^184^ ^]^ Moreover, based on recent studies, more than one stimulation method is generally utilized to achieve synergistic effects.

Yang's group combined pH and magnetic stimuli‐responsive release of drugs. In their study, magnetic and molecular targeting combined with a pH‐dependent slow release of Dox using FA‐functionalized GO–Fe_3_O_4_ (size < 200 nm) was observed.^[^ ^109^ ^]^ They first prepared GO–Fe_3_O_4_ via chemical deposition, then conjugated FA to Fe_3_O_4_ based on imide linkage with amino groups induced by 3‐aminopropyl triethoxysilane modification. The Dox was connected to GO through hydrogen‐bonding interactions and *π*–*π* stacking. They also indicated that the hydrogen bonding interaction between GO and Dox supports the pH‐dependent release. The combination of NIR and redox‐controlled release was also studied. Yin et al. synthesized hyaluronic acid‐decorated graphene oxide nanosheet (HSG) as a synergetic stimuli‐responsive drug delivery system (Figure 11c).^[^ ^130^ ^]^ Targeting of HSG to cancer cells was based on HA receptor, and accumulation of HSG in HA‐receptor overexpressed tumor cells was observed.

Then, NIR irradiation was applied to mediate endo/lysosome disruption for cytoplasm‐selective delivery. Finally, the redox‐triggered rapid release of DOX in the glutathione‐rich area enhanced pharmacologic action with the synergetic effect. In their study of biocompatibility and cytotoxicity, HSG was biodegradable and caused minimal collateral damage to normal tissues. Yang's group studied metastatic breast cancer targeting and drug delivery based on PEG–GO–NOTA–DOX conjugates.^[^ ^142^ ^]^ FSHR was expressed in various cancer types as an angiogenic marker. Their research used it for tumor targeting and stimulation of drug release. Conjugating PEG–GO–NOTA–DOX to an mAb against FSHR and labeled with ^64^Cu, made it possible to carry on specific drug delivery and present PET imaging. As for NIR stimulation, Wu et al. produced peptide–GO hybrid hydrogel for drug delivery and NIR‐triggered release.^[^ ^130^ ^]^ Weak interactions like hydrophobic and hydrogen bonds could be temporarily broken upon the NIR trigger to release the connected Dox. Regarding temperature‐triggered drug release, Tian's group synthesized GO–PEG–Ce6 to carry on photothermally enhanced PDT (Figure 11b).^[^ ^184^ ^]^ GO–PEG–Ce6 exhibited less ROS generation ability than free Ce6, but the former obtained better PDT efficacy due to increased cellular uptake.

Additionally, the synergistic effect of using an NIR laser with different wavelengths was confirmed in their studies, where an 808 nm NIR laser at a power density of 0.3 W cm^−2^ (360 J cm^−2^) was applied to increase the cellular uptake. A 660 nm laser at a low optical density of 50 mW cm^−2^ (15 J cm^−2^) was exposed to facilitate ROS release. Except for wavelengths, the effect of laser dose in a synergistic therapy pathway was also reported.^[^ ^181^ ^]^ The MSN‐Dox@GO‐Apt synthesized was an aptamer‐targeting and photoresponsive drug delivery system with multifunctions. Nucleolin‐specific targeting and real‐time indicator were achieved by the functionalization of Cy5.5‐AS1411 aptamer. GO, as a gatekeeper, prevented the loaded Dox from leaking and controlled the drug release under NIR irradiation. With increasing laser dose, the effect of PTT increased, and more Dox was released due to increased temperature, resulting in higher therapeutic efficacy. The optimal laser power density was 0.5 W cm^−2^, where MSN‐Dox@GO‐Apt (2.4 µg mL^−1^) killed more than 70% of MCF‐7 cells. Kim et al. combined NIR and glutathione (GSH) trigger mechanism based on a graphene composite, where branched polyethylenimine (BPEI) was functionalized on GO, followed by chemical reduction, then methoxypolyethyleneglycol was used to enhance the colloidal stability of BPEI–rGO, finally was the PEG functionalization and DOX conjugation.^[^ ^185^ ^]^ The DOX loading ratio of PEG–BPEI–rGO was found to be higher than that of GO, which may be attributed to the enhanced *π*–*π* stacking and hydrophobic interactions between rGO and DOX. NIR treatment would induce endosomal disruption and the proton sponge effect, which led the composite to escape from the endosome, and GSH triggered the release of DOX, where 20% decreased cell viability was found with NIR irradiation (Figure 11d).

### 5.3 Biosensor

Biosensors are devices sensitive to target biomolecules and can convert changes in the concentration of biomolecules into electrical signals^[^ ^186^ ^]^ or optical signals^[^ ^187^ ^]^ for detection and analysis. Due to high electrocatalytic activity, large surface area, good mechanical properties, and high stability, functionalized graphene materials offer unique advantages as sensing substrates for simple, fast, and ultrasensitive detection of biomolecular binding.^[^ ^10^ ^]^ For instance, the predicted mobility based on the first principle theory is calculated as high as 9.04 × 10^3^ cm^2^ V^−1^ s^−1^ for electrons at 300 K;^[^ ^188^ ^]^ the electron mobility is higher than traditional 2D semiconductors such as CrS_2_ (237 cm^2^ V^−1^ s^−1^), MoS_2_ (176–206 cm^2^ V^−1^ s^−1^), and WS_2_ (2010–2366 cm^2^ V^−1^ s^−1^);^[^ ^189^ ^]^ thus, it is beneficial for developing graphene‐based biosensors. Three categories of biosensors are discussed in this section. The first one is cyclic voltammetry (CV) measurement based on resistance changes;^[^ ^186^ ^]^ another method detects spectrum shifts.^[^ ^174^ ^]^ CV measurements can be further divided as (a) attaching specific enzymes to catalyze oxidation and changing resistance,^[^ ^186^ ^]^ (b) binding ligands like aptamers,^[^ ^104^ ^]^ antibodies,^[^ ^4^ ^]^ and peptides^[^ ^10^ ^]^ to adsorb targeting biomolecules and induce resistance changes specifically. The second is biosensor based on spectrum shifts methods; extrinsic NIR irradiation^[^ ^174^ ^]^ and intrinsic blue color reaction^[^ ^187^ ^]^ induce an enhanced variation in the spectrum after attaching to specific targets. Third, several more recent research demonstrated multifunctional graphene‐based biosensors that support functions including diagnosis, treatment,^[^ ^190^ ^]^ and multimode detection^[^ ^191^ ^]^ in vivo. In this section, graphene materials utilized for biosensing are classified as electric‐based biosensors, spectrum shift‐based biosensors, and multifunctional biosensors.

#### 5.3.1 Electric‐Based Biosensor

Since graphene demonstrates high electrocatalytic activity, applying graphene materials as electrodes for oxidase biosensors is widely investigated. Shan's group developed the first graphene‐based glucose biosensor using graphene/AuNPs/chitosan composites film.^[^ ^186^ ^]^ With the attachment of glucose oxidase (GOD), catalyzed oxidation of glucose consumed O_2_ and produced H_2_O_2_, which led to changes in current under different potentials. The glucose concentration can be derived from the changes in current. According to their results, the composites performed a good amperometric response to glucose with a linear range from 2 to 10 mm and a detection limit of 180 µm at 0.2 V. In another study of glucose sensing, Kang's group developed a biocompatible chitosan–GO–glassy carbon electrode (GCE),^[^ ^107a^ ^]^ where chitosan functionalization of GO improved dispersion and enzyme molecules were immobilized. As a result, GOD–GO–chitosan–GCE with high sensitivity (37.93 mA mm ^−1^ cm^−2^) and stability for glucose detection was developed.

**Figure 12:**
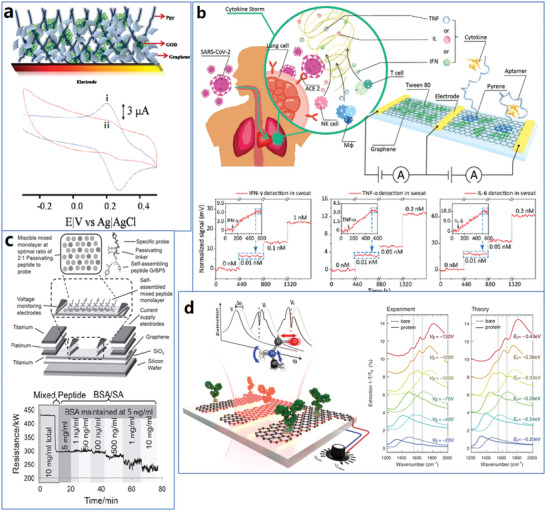
Functionalized graphene materials applied as biosensors. a) Synthesis and functionalization of Ppy–GOD–graphene and comparison of cyclic voltammetry of 100 mm glucose in pH 7.4 PBS buffer by using Ppy–GOD–graphene (i) and Ppy–graphene (ii). Reproduced with permission.^[^ ^192^ ^]^ Copyright 2010, American Chemical Society. b) Schematic of DGTFET biosensor for detecting biomarkers of cytokine storm syndrome caused by COVID‐19 and time‐resolved measurement of cytokines (IFN‐*γ*, TNF‐*α*, and IL‐6) at different concentrations in human sweat. Reproduced with permission.^[^ ^104^ ^]^ Copyright 2021, Wiley‐VCH. c) Schematic of the optimized self‐assembled GrBP5‐graphene FET and a sensogram where 25% bio‐GrBP5, 75% ss‐GrBP5 peptide mixture was added first, followed by BSA, which demonstrates no binding, and selectively capturing of streptavidin was detected when concentration is larger than 50 ng mL^−1^. Reproduced with permission.^[^ ^10^ ^]^ Copyright 2014, Wiley‐VCH. d) Schematic of mid‐IR induced plasmon resonance of graphene for detecting adsorbed proteins and the experimental and theoretical extinction spectra of the graphene nanoribbon array before and after protein bilayer formation. Reproduced with permission.^[^ ^174^ ^]^ Copyright 2015, The American Association for the Advancement of Science.

Similarly, Alwarappan et al. synthesized polypyrrole (Ppy) modified GCE as a biosensor to conjugate with GO–glucose oxidase.^[^ ^192^ ^]^ The porous structure of Ppy facilitated attaching GO on GCE, thereby presenting a stable and sensitive system for glucose detection with a good linear response in the range of 2–40 µm and a limit of detection of about 3 µm (**Figure** **12**a). Electrochemical DNA sensing based on graphene is another interesting field. The graphene‐based biosensor exhibited high sensitivity, selectivity, and low operation cost in detecting DNA sequences and further disease diagnosis. Direct oxidation of DNA is the simplest among all the electrochemical DNA sensing. Zhou's group developed a chemically reduced graphene oxide modified glassy carbon (CR‐GO/GC) electrode to separate signals of the four bases of DNA without the requirement for prehydrolysis.^[^ ^193^ ^]^ Additionally, the device directly detected single nucleotide polymorphism without hybridising additional labeling. Furthermore, ethanol detection was based on rGO/GC attached alcohol dehydrogenase.^[^ ^193^ ^]^

Apart from oxidation, simple bonding ligands with targets could also change resistance; the application of ligands bonding in virus detection attracted researchers’ attention. Graphene‐based biosensors could be applied in virus detection that achieves fast, accurate, and reliable detection of viruses early; for instance, SARS‐CoV‐2 antigen nucleocapsid protein detection was reported.^[^ ^194^ ^]^ Ménard‐Moyon et al. provided a comprehensive review of the 2D materials virus detection; readers interested in 2D materials except for graphene (Xenes, MXenes, MoS_2_, transition metal dioxides, gC_3_N_4_) are directed to this review.^[^ ^195^ ^]^ where interaction mechanisms between 2D materials and virus, functionalization methods of 2D materials, and progress in virus, proteins detections were involved. Except for the detection of nucleocapsid protein for virus detection, excessive release of cytokines of interferon (IFN), interleukin (IL), and tumor necrosis factor (TNF) were considered prognosis biomarkers for determining severe COVID‐19. Hao's group developed a dual‐channel graphene‐TWEEN 80 FET (DGTFET), with in vitro tests based on sweat, urine, and saliva of volunteers, and IFN‐*γ*, TNF‐*α*, and IL‐6 were detected in 7 min with limits of detection of 476 ×10^−15^, 608×10^−15^, and 611×10^−15^ m, respectively (Figure 12b).^[^ ^104^ ^]^ In their devices, graphene was functionalized with 1‐pyrenebutanoic acid succinimidyl ester and then attached with aptamers for specific cytokines binding. TWEEN 80 assisted in providing a passivation layer to eliminate the signals from the background. This DGTFET supports binding and twisting and is stable for several weeks. However, detecting mild or asymptomatic COVID‐19 still needs to be studied as their devices were only suitable for severe cases. Feng's group combined AS1411 aptamer with perylene tetracarboxylic acid functionalized GO to detect label‐free cancer cells.^[^ ^105^ ^]^ AS1411 exhibited binding affinity and specificity to overexpressed nucleolin of cancer cells; thus, normal and cancer cells were well distinguished. According to their results, the biosensor was reusable after being regenerated based on DNA hybridization techniques. Chae's group developed an oxygen‐plasma‐treated rGO surface as a biosensor to detect amyloid‐beta (A*β*) peptides.^[^ ^4^ ^]^ The oxygen‐plasma‐treated rGO sensor obtained better‐enhanced surface functionality, antibody immobilization, and sensing ability. Their results show that samples from AD and normal control (NC) subjects could be successfully identified in vitro. However, the time‐dependent effect of oxygen plasma treatment was a drawback (remained 46–51% after 6 h in vivo). Also, in AD diagnosis, Bungon et al. synthesized a graphene field‐effect transistor biosensor to detect Clusterin, an AD protein biomarker.^[^ ^58a^ ^]^ Cai's group combined PVP‐coated graphene with SBA‐15/horseradish peroxidase (HRP)/Ab_2_/BMIM·BF_4_ nanoparticles to enhance electrochemical immunoassay for detecting the BRCA1 gene. BRCA1, as an antioncogene, was genetically predisposed to breast and ovarian cancer cells; thus, it could be used for characterizing and screening patients against these two cancer types.^[^ ^135a^ ^]^ Under optimal conditions, BRCA1 could be detected in a wide range from 0.01 to 15 ng mL^−1^ and with a detection limit of 4.86 pg mL^−1^. Khatayevich et al. utilized engineered dodecapeptide GrBP5 to simultaneously passivate and functionalize graphene FET (Figure 12c).^[^ ^10^ ^]^ The SS‐GrBP5 mutant (sequence of SS‐IMVTESSDYSSY) employed was self‐assembled into ordered monolayers on graphene sheets, and a hydrophilic surface was obtained. Due to the contact angle similar to antifouling systems demonstrated by other groups, they hypothesized that self‐assembled peptide monolayers also obtained the antifouling ability and thus decreased the nonspecific bonding of peptides in BSA. According to their results, the device selectively detected streptavidin against a serum albumin background with a detection limit of 50 ng mL^−1^.

#### 5.3.2 Spectrum Shift‐Based Biosensor

Rodrigo's group studied graphene's mid‐IR‐induced plasmon resonance to detect adsorbed proteins.^[^ ^174^ ^]^ As the electromagnetic field is concentrated at the graphene edges, the IR beam can enhance the light interactions of attached protein molecules and present a plasmon resonance spectral shift. Different proteins obtained varied vibrational fingerprints; thus, proteins could be distinguished based on the plasmon resonance spectral shifts (Figure 12d). According to their in vitro tests with recombinant protein A/G and goat antimouse immunoglobulin G (IgG), graphene as a biosensor exhibited higher sensitivity than traditional metallic plasmonic sensors. Song et al. detected the concentrations of glucose and H_2_O_2_ using the colorimetric method instead of CV measurements.^[^ ^187^ ^]^ An intrinsic peroxidase catalytic ability GO‐COOH was obtained to catalyze the peroxidase substrate 3,3,5,5‐tetramethylbenzidine (TMB) in the presence of H_2_O_2_ and then produced a blue color. This catalytic reaction was dependent on pH, temperature, and H_2_O_2_ concentration. Their results showed that pH 4.0, 35 °C, and 150 mm were the optimal environments. Additionally, it was confirmed that the linear range of glucose detected was from 1 × 10^−6^ to 2 × 10^−5^ mol L^−1^, with a detection limit as low as 1 × 10^6^ mol L^−1^. Though the catalysis of GO‐COOH and HRP were both ping‐pong mechanisms (reacts with the first substrate, then releases the first product before reacting with the second substrate), GO‐COOH exhibited higher catalytic activity to TMB due to accelerated electron transfer.

#### 5.3.3 Multifunctional Biosensor

Some recent studies revealed the development of in vivo biosensors based on graphene materials for brain signals detection and electrical pulse delivery for treatment. Park et al. developed a graphene‐based electrical device that records and passes electrical signals into cortical areas for diagnosis and electrical stimulations embedded in a subset of the graphene multichannel array for suppressing epileptic seizures after detecting the epileptic signals.^[^ ^190^ ^]^ The graphene was prepared via CVD on Cu foil, then transferred to SU‐8 epoxy film (5 µm thickness) with PMMA supporting layer, followed by photolithography and oxygen plasma etching; finally, nitro and Ag were doped to improve the electrical conductivity. Compared with penetrating electronics that lack large‐scale, real‐time, and safe recording/stimulation, this graphene‐based electrode took advantage of low electronic noise, good biocompatibility, mechanical flexibility, and remarkable optical properties. Graphene with 1, 2, and 4 layers was tested, where increased layer provided lower impedance and higher capacitance, 4‐layer graphene obtained the best performance with ≈10^3^ Ω with a frequency of 20 Hz and capacitance of 326.7 F g^−1^.^[^ ^190^ ^]^ In vivo tests with mice models demonstrated the efficiency of such electrodes, and it is supposed that this graphene‐based electrode could be used for potential applications in tinnitus, Parkinson's disease, Huntington's disease, depression, and schizophrenia treatment. In another study, two models of imaging were combined based on a graphene biosensor, where Kuzum et al. applied an Au‐doped graphene electrode achieving simultaneous electrical recording for interictal and ictal activity detection in population discharges and fast population spikes with durations less than 5 ms and calcium imaging using two‐photon microscopy based on an 840 nm femtosecond laser pulse to excite Oregon Green BAPTA‐1 AM (OGB‐1).^[^ ^191^ ^]^ The 50 µm electrode obtained a 5‐to‐6‐fold improvement in SNR and a 100‐fold decrease of electrical noise in electrophysiology. Due to the accessibility of the electrode to the brain, both good temporal and spatial resolution were brought. While the two‐photon microscopy was advantageous in providing less optical toxicity compared with confocal microscopy, the imaging depth was also improved according to the test of hippocampal slices from mouse brains. The in vivo multimode graphene‐based biosensors nowadays are mainly utilized.

### 5.4 Tissue Engineering

Tissue engineering aims to control the proliferation and differentiation of cells to form artificial organs in vitro, which can provide substitutes for severely injured organs and present potential solutions for diseases with unknown mechanisms.^[^ ^1^ ^]^ Most of the studies are related to tissue regeneration. Researchers aim to develop a method that overcomes the limitations of the lack of organ donors, disease transmission, and immune reactions using regenerated tissue produced with biocompatible scaffolds.^[^ ^116^, ^117^, ^118^, ^119^, ^120^, ^121^, ^122^, ^123^, ^124^, ^125^, ^126^, ^127^, ^128^, ^129^, ^130^, ^131^, ^132^, ^133^, ^134^, ^135^, ^136^, ^137^, ^138^, ^139^, ^140^, ^141^, ^142^, ^143^, ^144^, ^145^, ^146^, ^147^, ^148^, ^149^, ^150^, ^151^, ^152^, ^153^, ^154^, ^155^, ^156^, ^157^, ^158^, ^159^, ^160^, ^161^, ^162^, ^163^, ^164^, ^165^, ^166^, ^167^, ^168^, ^169^, ^170^, ^171^, ^172^, ^173^, ^174^, ^175^ ^]^ Materials used for tissue engineering need to be biocompatible as well as without inducing immune reactions (not toxic). Graphene materials can be applied as substrates due to their unique electrical and mechanical properties. After a suitable functionalization, these substrates can mimic the nanometer dimensions of extracellular matrices (ECMs) and create predefined structures to assist the growth of cells. This section is divided based on the structural features, where substrates with porous, flat, or fibrous structures are discussed.

#### 5.4.1 Porous Substrates

Porous substrates synthesized with graphene and polymers take advantage of keeping moisture for cell proliferation, enhancing the transport of nutrients and proteins, and ensuring good mechanical properties.^[^ ^116^ ^]^ Sarkar et al. investigated hydrogel for bone‐related biomedical engineering.^[^ ^3^ ^]^ They synthesized poly(acrylic acid) (PAA) hydrogel cross‐linked by GO to improve the toughness, extensibility, and stress distribution. It was noted that GO connected with PAA both via covalent cross‐linking (free‐radical polymerization reaction) and physical cross‐linking (vdW, ion–dipole, electrostatic interaction, etc.) were responsible for achieving high toughness (7124 kJ m^−3^) and extensibility (elongation at break 4078%). However, in vitro cell tests for tissue regeneration were not conducted. Ma et al. synthesized nanocomposite sponges of sodium alginate (SA)–GO–PVA based on the freeze–thawing cyclic process and freeze‐dried molding for wound dressing application.^[^ ^124^ ^]^ With GO concentration at less than 2 wt%, the sponges obtained a homogeneously porous and interconnected network structure to offer proper water absorption, breathability, and mechanical properties beneficial for cell proliferation.

**Figure 13:**
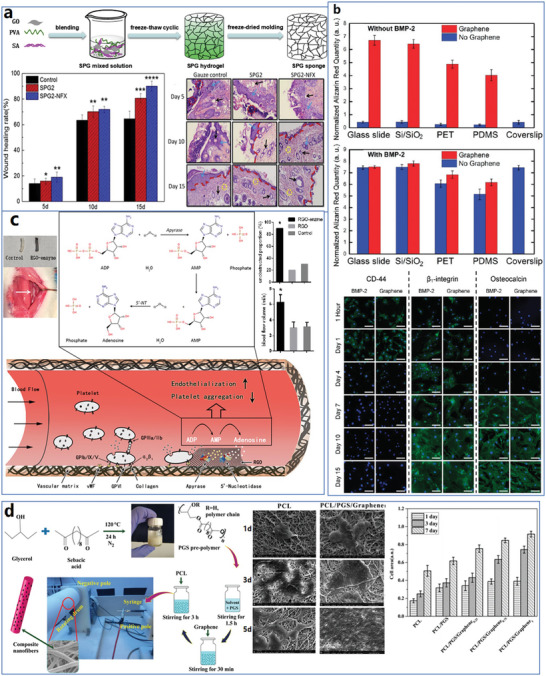
Application of substrates synthesized with functionalized graphene materials in tissue engineering. a) Synthesis of SA–GO–PVA nanocomposite sponges, wound healing rate, and H&E staining section of mouse skin evaluated at different times after treatment with gauze control, SPG2, and SPG2‐NFX. Reproduced with permission.^[^ ^124^ ^]^ Copyright 2019, Elsevier Ltd. b) Cells grown in different substrates with and without BMP‐2, where coverslips are used as a reference, and immunostaining images of cells growth on Si/SiO_2_ substrates either with BMP‐2 or coated with graphene. CD‐44, *β*1‐integrin, and OCN are makers for stem cells, cell–substrate adhesion, and bone cells, respectively. Reproduced with permission.^[^ ^58b^ ^]^ Copyright 2011, American Chemical Society. c) Mechanism of rGO–enzyme coated TEBV for suppressing platelet aggregation, and the evaluation results of unobstructed proportion, blood flow volume after seven days. Reproduced with permission.^[^ ^175^ ^]^ Copyright 2017, American Chemical Society. d) Synthesis of graphene‐embedded PCL/PGS nanofibrous scaffolds, FESEM images of cultured human cardiac myocytes cells (HCMs) on bare PCL and 1 wt% graphene‐embedded PCL/PGS nanofibrous scaffolds, and the fraction of covered area by cells on substrates with different proportions of graphene. Reproduced with permission.^[^ ^107b^ ^]^ Copyright 2021, Wiley Periodicals LLC.

Additionally, they attached norfloxacin (NFX) for its antibacterial property; in vitro tests demonstrated a gradual release of the loaded NFX effectively against *Escherichia coli* and *Staphylococcus aureus*. Based on the hemolysis tests and CCK‐8 assay, they confirmed that the complex was nonhemolytic and could promote cell proliferation. Finally, according to in vivo tests, the SA–GO–PVA–NFX complex effectively prevented inflammation in the epithelial tissues, which successfully healed the wound and reduced scar formation. The complex could be gradually degraded (**Figure** **13**a). Depan et al. studied the proliferation and growth of osteoblasts on GO–chitosan substrates.^[^ ^135b^ ^]^ The synthesized GO–chitosan scaffolds showed hydrophilic surfaces, high water retention ability, and high interconnectivity. According to their results, GO–chitosan scaffolds with porous structures improved the movement of growth factors and cells, provided enhancement of cell attachment and proliferation, as well as showed stability against enzymatic degradation.

#### 5.4.2 Flat Substrates

Flat substrates are mainly used for cell proliferation^[^ ^196^ ^]^ and differentiation,^[^ ^9^, ^58^ ^]^ while single‐layer and small lateral‐size substrates can be applied in artificial organs to pacify organ transplant rejection.^[^ ^175^ ^]^ Stem cells could differentiate into cells with different characteristics based on suitable growth factors and tissue‐regenerative inducers. Graphene materials are found to take the place of growth factors and accelerate the differentiation of cells together with different tissue‐regenerative inducers.^[^ ^58b^ ^]^ Based on different studies, the differentiation‐enhancement property of graphene was related to the usage of tissue‐regenerative inducers and the type of stem cells and substrate materials.^[^ ^9^, ^58^ ^]^ Nayak et al. transferred graphene synthesized using the CVD method to substrates of PDMS, PET, glass slide, and Si/SiO_2_.^[^ ^58b^ ^]^ Then hMSCs were cultured to observe whether hMSCs could differentiate into bones with the osteogenic medium that contained dexamethasone and lacked growth factors such as BMP‐2. Their results confirmed that the bone cells were detected with graphene‐based substrates, and the differentiation rate was similar to growth factors (Figure 13b).

Graphene showed good cell adhesion and charge transfer property, facilitating the activation of the intracellular signaling system. Thus, graphene could take the place of growth factors. However, if dexamethasone was provided on bare substrates without graphene, hMSCs differentiated into different cells at various bare substrates. Neurons were detected on uncoated PDMS, muscle cells were observed on PET, while stem cells on glass slides and Si/SiO_2_ did not differentiate. The proliferation and differentiation of mouse neuroblastoma Neuro 2A (N2a) cell lines were studied using graphene synthesized with CVD methods, and retinoic acid was utilized as a nerve inducer.^[^ ^9^ ^]^ Compared with glass substrates, N2a cells proliferate better on graphene transferred to Si/SiO_2_, and they observed enhanced differentiation into neuron‐like cells with graphene.

Additionally, poly‐d‐lysine (PDL), a specific cell adhesion factor, could improve the proliferation of N2a cells on any substrate. However, PDL exhibited better effects on graphene due to higher affinity, attributed to the differences in the arrangement between graphene and glass. The affinity of cells and substrates is supposed to be important in tissue engineering. The difference in affinity explains why graphene can take the place of growth factor, while cells on glass or Si/SiO_2_ are difficult to proliferate and differentiate. The different behaviors of stem cells are probably related to electron transfer, considering that the behaviors of cells are affected by membrane potentials. Different substrates provide varied electron transfer efficiency and, thus, different membrane potentials.^[^ ^9^, ^58^ ^]^ Changes in membrane potentials may influence the connection between dexamethasone and DNA sites. When connecting at sites of enhancers, a specific RNA expression and cell differentiation may be induced, while silencer site connection may forbid the differentiation of cells.

Except for stem cells, proliferation in normal cells was also examined. Liu's group developed a PVA–GO–citicoline sodium–lanthanum (PVA/GO–CDPC–La) film for wound dressing based on the solution intercalation method.^[^ ^196^ ^]^ The zwitterionic citicoline (CDPC) was utilized to modify GO to improve dispersibility, provide adsorption sites for La^3+^ ions and improve the antibacterial property. They found that the complex exhibited good wound exudate absorption and moisture‐retaining abilities. According to in vitro tests, it illustrated good biocompatibility, and cells proliferated well without bacterial proliferation. Finally, the in vivo tests proved that the product healed wounds and formed a mature epidermal architecture in two weeks.

In vitro and in vivo tests of implants based on graphene materials were also reported in recent research, especially for bone and dental applications. Rikhari et al. developed polypyrrole (PPy)/GO composite coating on Ti implants. The GO was synthesized based on a modified Hummer's method; GO was then dispersed in aqueous oxalic acid and pyrrole solution with ultrasonication, and the composite coating was achieved by a one‐step electropolymerization.^[^ ^197^ ^]^ The PPy/GO with GO of 5 wt% was found to be nonuniformly coated on Ti, with a cauliflower‐like morphology, which benefited from providing larger surface roughness (208 nm) and wettability (26° water contact angle) for cell viability (94% in 7 days) in biomedical applications compared with those with 1 and 3 wt% GO. Adding GO was also proved to increase the ability against corrosion due to the creation of a reinforcement network in PPy that protect the Ti metal, where both pore and charge transfer resistance increased with switching potential from −0.55 to 1.25 V. Adhesion test demonstrated a less than 5% removal of the coatings for lattice edges (4B class). The immersion test verified that hydroxyapatite (HAP, a compound with similar inorganic components to bones) could be grown on the PPy/GO–Ti composite. Their research demonstrated a potential direction of modifying and promoting the performance of Ti‐based on graphene materials for developing bone implants with enhanced stability, osseointegration and cell attachments. It is interesting to find a successful in vivo application of graphene materials as dental implants. Shin et al. proposed a method to do surface modification of the sandblasted, large‐grit, and acid‐etched (SLA) Ti disk (in vitro) and implant (in vivo) based on rGO. The roughness was increased while the contact angle of water decreased, which benefited the cell attachment and proliferation compared with the immobilization of recombinant human bone morphogenetic protein‐2 (rhBMP‐2) on Ti.^[^ ^198^ ^]^

Additionally, in vitro tests based on hMSCs demonstrated osteogenic differentiation, adhesion markers including alkaline phosphatase (ALP), osteocalcin (OCN), osteopontin (OPN), and vinculin were enhanced by rGO without any osteogenic factors, probably due to the ability to absorb exogenous proteins. In vivo study implanted the SLA Ti‐rGO into a beagle dog's mandible. According to the results from the removal torque test, histomorphometric study, and µ‐CT images, the osseointegration and dental tissue regeneration were enhanced by the rGO group compared with others 8 weeks after the surgery. Their research is comprehensive, and promising and is believed to obtain great potential in applying dental or orthopaedic implants.

In terms of antirejection when fabricating artificial organs, Huo et al. examined rGO‐based dual‐enzyme (apyrase and 5′‐nucleotidase (5′‐NT)) substrates for antiplatelet function and endothelialization ability of tissue‐engineered blood vessels (TEBVs).^[^ ^175^ ^]^ The enzymes on substrates facilitated the cascading reactions of catalyzing ADP to AMP and AMP to adenosine, which reduced or reversed the platelet aggregation induced by ADP and suppressed thrombosis. Furthermore, adenosine produced in the reactions accelerated the endothelialization of TEBVs by controlling and optimizing the cellular energy metabolism and microenvironment without triggering inflammation. Even after losing the activity of the rGO–enzyme complex, the antithrombotic function of TEBV could be retained (Figure 13c). According to their in vivo and in vitro results, these TEBVs grafted with rGO‐based dual‐enzyme substrates obtained a patency rate of 90% after seven days.

#### 5.4.3 Fibrous Substrates

Fibrous substrates mainly regenerate cardiac tissues since unique structures provide better electrical signal‐conducting behavior. Fakhrali et al. synthesized graphene‐embedded poly(caprolactone) (PCL)/PGS‐based nanofibrous scaffolds for cardiac tissue regeneration.^[^ ^107b^ ^]^ The electrospinning process produced PCL/PGS nanofibers, and graphene with 1 wt% was added to generate the relative electrical conductivity necessary for the conduction of electrical impulses in cardiac tissue (Figure 13d). According to their results, the hydrophilic nanofibrous scaffolds (diameter 343 ± 68 nm) exhibited good elastic modulus (11.41 ± 3.58 MPa). MTT assay showed that the composite is biocompatible and nontoxic, with cell viability larger than 98% after seven days.

As bacterial infections are an obstacle to the long‐term and stable application of biomedical implants or artificial organs, all three categories of graphene‐based materials for tissue engineering require antibacterial properties if applied for in vivo or in vitro cultivation.^[^ ^199^ ^]^ Mohammed et al. did a comprehensive review of the antimicrobial mechanism and effectiveness of graphene‐based materials.^[^ ^200^ ^]^ Antibiotic‐resistant pathogens may lead to implants removal long‐term after surgery; However, metallic ions such as silver and nitric oxide (NO) were proposed to be antibacterial, and the efficiency and safety required to be improved as pathogens may inhibit ions penetration based on outer membrane modification or bind and pump out metals via efflux pump, additionally, the high dose may induce side effects such as irreversible pigmentation or inflammatory response. Therefore, graphene‐based materials were proposed to be applied due to their intrinsic antibacterial activity targeting bacteria, fungi, and viruses. The antibacterial mechanisms were mainly attributed to the five cytotoxicities induce routes, as discussed in Section 4.1.1, where mechanisms of cell starvation in GO wrapping and passive self‐killing of cells by gene toxicity were still unclear.^[^ ^16^, ^139^, ^200^ ^]^ Lateral size, layers, particle shape, functional groups, and aggregation or dispersion of graphene materials were proposed as factors that influence antibacterial activity. Neither too large nor small size and thickness led to lower efficiency. It is suspected that the size and thickness factors induced different efficiencies in the routes that led to bacterial death. Thus, there would be an optimal size and thickness for specific bacteria determined by the surface energy of graphene materials, which requires further study based on experiments and molecular dynamics studies. Smooth‐top‐side and rough‐bottom‐side shape graphene obtained better antibacterial activity;^[^ ^200^ ^]^ however, the cytotoxicity of these materials for normal cells or systems containing both normal cells and bacteria was not mentioned in the tissue engineering studies. Aggregation of graphene and lower oxygen‐contained groups induced decreased efficiency in antibacterial ability, where decreased surface energy was the essence. Therefore, successful tissue engineering studies based on graphene materials, especially the long‐term and stable application, still require studies combining theoretical mechanisms and experimental phenomena.

### 5.5 Photothermal Therapy

Biocompatible graphene materials with high‐laser absorption and thermal conductivity are suitable for PTT.^[^ ^1^, ^44^ ^]^ PTT is performed by delivering graphene materials specifically into the targeted nidus and utilizing the laser absorption and thermal conversion behavior of graphene materials to ablate the nidus under NIR irradiation.^[^ ^2^, ^8^ ^]^ Moreover, damage to surrounding normal tissues should be negligible in an optimal PTT. According to recent studies, PTT techniques are classified as basic targeting PTT and enhanced PTT.

#### 5.5.1 Basic Targeting PTT

**Figure 14:**
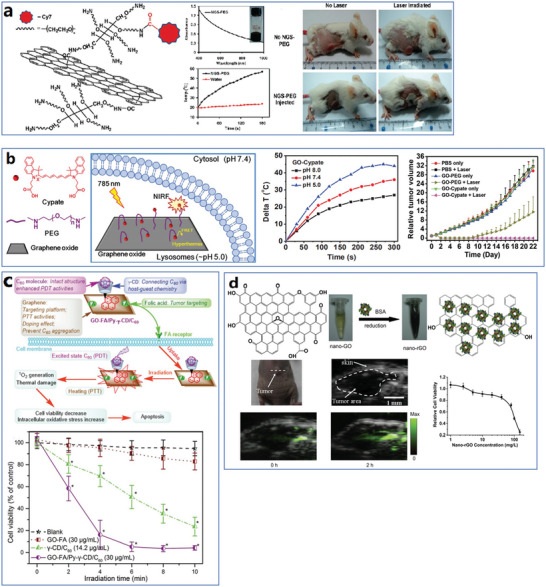
Application of functionalized graphene materials in PTT. a) Structure and laser absorption behavior of PET–GO–Cy7 (0.05 mg mL^−1^), temperature increase curves with 808 nm laser at a power density of 2 W cm^−2^, and photos of mice bearing tumors provided with various treatment conditions, ablation of tumors occur only with both NIR irradiation and PET–GO–Cy7. Reproduced with permission.^[^ ^2^ ^]^ Copyright 2010, American Chemical Society. b) Mechanism of enhancing photothermal performance mediated by pH‐dependent FRET, temperature increase curves of GO–Cypate (16.5 µg mL^−1^ GO, and 5.0 µg mL^−1^ Cypate) in buffers at different pH with 5 min NIR irradiation, and comparison of the therapeutic effects. Reproduced with permission.^[^ ^8^ ^]^ Copyright 2014, Wiley‐VCH. c) Mechanism of the synergistic effects of PDT/PTT using GO‐FA/*γ*‐CD/C_60_ and comparing the therapeutic effects. Reproduced with permission.^[^ ^201^ ^]^ Copyright 2017, Royal Society of Chemistry. d) Synthesis and application of BSA–rGO for the combination of PAI and PTT. Reproduced with permission.^[^ ^49b^ ^]^ Copyright 2013, Elsevier Ltd.

In the early stages of research, nidus‐targeting in PTT mainly relies on passive tumor targeting or attaching ligands to graphene materials without any control or enhancing strategies. Yang et al. covalently functionalized GO with PEG and conjugated Cy7 dye covalently to PET‐GO for labeling.^[^ ^2^ ^]^ The composite exhibited highly efficient tumor passive targeting and low retention in RES. The intravenous injection of 200 µL PEG–GO at 2 mg mL^−1^ (20 mg kg^−1^) to mice‐bearing tumors was tested, with the exposure of 808 nm NIR laser at a power density of 2 W cm^−2^ every two days, and the tumor ablated within one week without showing signs of regrowth in 40 days. Histology analysis demonstrated that 10–50 nm single‐ or double‐layered PET–GO would not cause significant toxicity to normal tissues. Also, mice were confirmed to remain healthy for three months after treatment (**Figure** **14**a). Studies showed that rGO is more likely to be applied in PTT due to better laser absorption than GO. Robinson et al. functionalized rGO with PEG noncovalently, and sixfold higher NIR absorption than PEG–GO (covalently functionalized) was observed.^[^ ^76^ ^]^ Then, aiming at specific internalization of U87MG cancer cells, a targeting peptide bearing Arg–Gly–Asp (RGD) motif was attached to rGO–PEG (mean size of 18.8 nm). Their in vitro tests based on 15 W cm^−2^ NIR light for 8 min showed that U87MG cells were destroyed with 6.6 mg L^−1^ rGO–PEG–RGD, while control groups obtained ≈100% viability. Also, they proposed that rGO would be a lower‐cost NIR photothermal agent compared with Au nanoparticles and CNT.

#### 5.5.2 Enhanced PTT

The targeting and controllable PTT provide a more efficient treatment to focus sites than basic targeting PTT. The synergistic effect of combining PTT with other treatment techniques has also attracted much attention. Guo et al. synthesized pH‐responsive cyanine‐grafted PEG–GO for FRET‐enhanced PTT.^[^ ^8^ ^]^ As an organic NIR agent exhibiting near‐infrared fluorescence (NIRF), cyanine may produce fluorescence when treated with NIR laser via a radiative transition instead of a nonradiative transition, decreasing photothermal transmission efficiency. It is found that the NIRF would be quenched via FRET if cyanine is aggregated on GO, and the photothermal transmission efficiency can be promoted. Therefore, a pH‐dependent mechanism of the complex conformation was investigated to apply the FRET, where cyanine molecules tend to be dislocated from the PEG–GO surface at pH 8. The decrease in pH led to the aggregation of cyanine molecules on GO (Figure 14b). In their in vitro experiment based on murine 4T1 breast cancer cells, it was confirmed that the composites were internalized by lysosomes (decreased pH from 7.4 to 5.0) via clathrin‐mediated endocytosis, the FRET was induced with NIR laser exposure under a decreased pH, and significant tumor ablation was observed. According to their in vitro tests, the composite induced significant 4T1 cells cytotoxicity with an IC_50_ value of 6.0 µg mL^−1^ using an NIR laser for 3 min at 1.5 W cm^−2^. In addition, in vivo tests by intravenous administration of 7.5 mg kg^−1^ composites showed an accumulation of the composite in the tumor due to an enhanced permeability and retention effect (EPR) in the liver and kidney after 24 h.

Moreover, it was reported that the NIRF noise from normal tissues was relatively low after five days. At the same time, a significant signal was obtained from tumor sites, which indicated PEG–GO–cyanine clearance from normal tissue but is retained in tumors. If without NIR laser, no significant cytotoxicity was observed in 22 days, but with NIR laser of 785 nm, 5 min, and 1.0 W cm^−2^, there were necrosis and regression of tumors after two days and without regrowth after 22 days. Hu et al. fabricated GO‐FA/*γ*‐CD/C60 nanohybrid for the synergistic therapy of PTT and PDT.^[^ ^201^ ^]^ They first functionalized GO with FA and bonded with *γ*‐Cyclodextrin (CD) via *π*–*π* interaction. Finally, the cavities on the GO‐FA/*γ*‐CD surface allowed C60 to be loaded based on host–guest chemistry (Figure 14c). Host–guest chemistry with GO‐FA/*γ*‐CD could keep the integrity and photon response of C_60_. At the same time, it hindered the aggregation as well as reduced toxicity. As a multifunctional nanohybrid, GO provides stability and biocompatibility. FA assists in targeting tumor cells and *γ*‐CD was utilized as a host molecule for host‐guest chemistry with C60 and C60‐induced PDT and enhanced PTT. During the in vitro test of HeLa cells under Xe lamp irradiation (2 W cm^−2^) for 4 min, cell viability reduced to 16.2% even at a low concentration (30 µg mL^−1^) of this composite (Figure 14c). PTT could also be combined with imaging. Li's group synthesized thioflavin‐S (ThS)‐modified GO for photothermal treatment of AD in mice CSF.^[^ ^106^ ^]^ Their results showed GO‐ThS dissociated amyloid deposits in buffer and mice CSF and protected cells from A*β*‐related toxicity upon NIR irradiation.

Additionally, the fluorescence change could monitor the disaggregation of A*β* fibrils. Sheng et al. utilized BSA as a reductant for GO reduction. BSA was further adsorbed on rGO via hydrophobic and *π*–*π* interaction.^[^ ^49b^ ^]^ The BSA–rGO synthesized could be applied for both PAI and PTT in vivo. They carried out both in vitro tests on MCF‐7 breast cancer cells and in vivo tests based on mice models using BSA–rGO. In vitro test demonstrated that 0.1 mg mL^−1^ rGO–BSA could increase the temperature from 25 to 55 °C in 5 min with a diode‐laser (808 nm, 0.6 W cm^−2^) (Figure 14d), facilitating the tumor ablation effect. In vivo tests by administrating 1 mg mL^−1^ rGO–BSA intravenously showed a significant PA signal in the tumor region compared to normal tissues due to the EPR effect. When treated with the same laser, the temperature in the tumor tissue increased from 37 to 53 °C and induced tumor ablation successfully (Figure 14d). They also observed no cytotoxicity on MCF‐7 cells, even with a high concentration of 40 mg mL^−1^. The histological analysis results were also normal.

### 5.6 Gene Delivery

Apart from the delivery of drug molecules, graphene materials could also behave as carriers in gene delivery. The interactions between graphene materials and nucleic acids reported in previous research were mainly described in three unique modes 1) preferential adsorption of single‐strand DNA (ssDNA) over double‐strand DNA (dsDNA),^[^ ^1^ ^]^ 2) prevention effects of nuclease enzymes based on steric protection of adsorbed nucleotide,^[^ ^202^ ^]^ and 3) small graphene layers could intercalate to DNA and induce DNA scission.^[^ ^132^ ^]^ Also, interaction energies between graphene and DNA nucleobases were reported as thymine (T) < cytosine (C) < adenine (A) < guanine (G) in aqueous solutions.^[^ ^133^ ^]^ Apart from basic interaction mechanisms, more focus was directed toward exploring applications. Gene delivery based on graphene materials could be applied for both the detection of diseases based on ssDNA^[^ ^176^ ^]^ and the gene therapy relying on plasmid DNA (pDNA) transfection^[^ ^117^ ^]^ or siRNA‐induced gene silence,^[^ ^162^ ^]^ where challenges from potential genotoxic effects of graphene materials to normal cells can be overcome by applying suitable functionalization methods.^[^ ^132^ ^]^ This section introduces gene delivery techniques from the diagnosis and therapy aspects.

#### 5.6.1 Gene Delivery for Diagnosis

**Figure 15:**
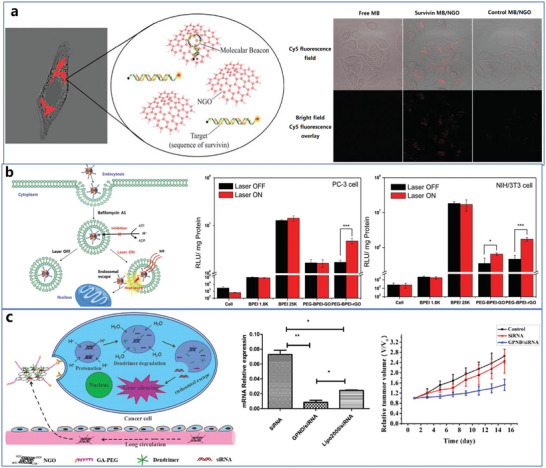
Application of functionalized graphene materials in gene delivery. a) Mechanism of GO delivery of MB into HeLa cells to detect survivin mRNA and the confocal fluorescence microscopy results. Reproduced with permission.^[^ ^176^ ^]^ Copyright 2010, Royal Society of Chemistry. b) Schematic representation of NIR triggered endosome escape of BPEI–rGO complex attached pDNA to enhance and evaluate the transfection efficiency. Reproduced with permission.^[^ ^125^ ^]^ Copyright 2013, Wiley‐VCH. c) Schematic illustration of uptake, release, and gene silence induced by GPGD–anti‐VEGFa siRNA and the evaluation of VEGFa mRNA expression and growth curves of liver tumor tissues. Reproduced with permission.^[^ ^162^ ^]^ Copyright 2019, Elsevier B.V.

Fluorescence imaging based on graphene materials functionalized with ssDNA or other molecular beacons (MB) that brings a terminal‐labeled fluorophore can be applied in diagnosis. When the composites combine with the target DNA or RNA of nidus cells, viruses, and germs,^[^ ^131^, ^176^ ^]^ the stronger combination between ssDNA/MB and target DNA or RNA terminates the fluorescence quenching effects and reactivates fluorescence. However, the fluorescence signal is not strong enough to penetrate deep tissues. Thus, gene delivery for diagnosis is only available in vivo. Lu et al. proposed the protection of oligonucleotides from enzymatic cleavage during cellular delivery based on GO, ensuring the stability of target DNA detection.^[^ ^176^ ^]^ In their research, MB was adsorbed on GO (size < 100 nm) noncovalently, which not only protected MB from cleavage based on the steric hindrance effect but also enhanced cellular delivery due to high GO cell uptake (**Figure** **15**a). The fluorescence of MB that brought a terminal‐labeled fluorophore was quenched when connected with GO. After hybridizing with target mRNA or DNA, MB was released from GO and restored fluorescence to provide cellular‐florescence imaging.

Additionally, the toxicity of this composite is negligible. Even with a concentration of 100 mg L^−1^, cell viability was close to 100%. Xu's group utilized a self‐assembling strategy to synthesize multifunctional hydrogels from GO sheets and DNA.^[^ ^131^ ^]^ According to their results, due to strong *π*–*π* stacking, electrostatic and hydrophobic interactions between ssDNA and GO, the GO/DNA‐composite‐self‐assembled hydrogel (GO/DNA SH) exhibited good mechanical strength and high stability. The complex also exhibited a self‐healing property where dsDNA is re‐formed with excessive ssDNA in SH during the cooling process and self‐assembled to synthesize GO/DNA SH when heating recurred. The GO/DNA SH is also supported with loading dye for detection based on the electrostatic interaction between positively charged safranine O and negatively charged GO sheets. Sheng's group proposed an aptamer‐based sensing method based on PVP‐coated GO and a fluorescently tagged aptamer,^[^ ^203^ ^]^ similar to the aptamer biosensor discussed in Section 5.3. Du et al. reported detecting target DNA (D‐vasopressin (D‐VP)) and single nucleotide polymorphisms using a conjunction of split aptamer on graphene mesoporous silica gold NP hybrids.^[^ ^204^ ^]^ The complex obtained high sensitivity (10 fm) and an excellent detection limit of 5 ng mL^−1^ without affinity for the enantiomer of L‐VP. Compared with surface‐enhanced Raman spectroscopy and matrix‐assisted laser desorption/ionization mass spectrometry, this technique is more cost‐ and time‐effective when applied as a chiral selector.

#### 5.6.2 Gene Delivery for Therapy

The major obstacle in this area is developing a nonviral‐based, safe, and efficient gene delivery vehicle; in this regard, graphene could be a good choice.^[^ ^117^ ^]^ The transfection effect of pDNA delivery is a typical example of gene therapy, where graphene materials functionalized with cationic polymers are utilized. Feng's group synthesized cationic GO–PEI (10 and 1.2 k molecular weight) with noncovalent electrostatic interaction, further bonded with anionic pDNA via a layer‐by‐layer assembly process for intracellular transfection of the enhanced green fluorescence protein (EGFP) gene in HeLa cells.^[^ ^117^ ^]^ The cytotoxicity of GO–PEI‐10 k was lower than free PEI‐10 k but remained similar to EGFP transfection efficiency, while GO–PEI‐1.2 k obtained much better EGFP transfection than PEI‐1.2 k. They proposed that suitable molecular weight and N/P ratio were needed to reduce cytotoxicity and increase transfection efficiency. Recent investigations^[^ ^118^, ^162^, ^205^ ^]^ also considered the size control of GO and other additional functionalization methods. Teimouri et al. compared the transfection efficiency and cytotoxicity of GO conjugation with alkylated derivatives of cationic polymers, including PEI and PPI, and PAMAM.^[^ ^118^ ^]^ The three cationic polymers were connected to GO based on the surface carboxyl group, glycine, and spermidine. They further utilized a plasmid DNA of enhanced green fluorescent protein to evaluate the transfection efficiency and MTT assay for cytotoxicity evaluation. Compared with bare cationic polymers, the cytotoxicity of all three complexes decreased notably, while the transfection efficiency was significantly enhanced.

Additionally, carrier/plasmid (C/P) weight ratios also affected the toxicity and transfection efficiency. Therefore, suitable functionalization methods and the C/P ratio need to be considered. Similarly, Liu's group synthesized GO–oleate–PAMAM hybrids for gene delivery.^[^ ^205^ ^]^ By the adsorption of oleic acid and then covalently linked PAMAM, the complex obtained good dispersity, biocompatibility, and high transfection efficiency. More recent research in this area also paid attention to synergistic effects. Kim et al. studied photothermally controlled gene delivery by conjugating BPEI and rGO via a hydrophilic PEG spacer.^[^ ^125^ ^]^ The PEG–BPEI–rGO complex attached pDNA via electrostatic interaction was confirmed to be stable in the physiological environment. They conducted an in vitro gene transfection study with PC‐3 and NIH/3T3 cells. It was demonstrated that the complex induced negligible cytotoxicity and enhanced gene transfection efficiency when treated with an NIR laser (Figure 15b). Bafilomycin A1, a proton sponge effect inhibitor, assisted NIR irradiation in accelerating endosome escape and improving transfection efficiency.

Besides the connection of pDNA to PEI–GO, small interfering RNA (siRNA) with therapeutic functions could also be delivered. Qu et al. reported a system consisting of glycyrrhetinic acid (GA), GO–PEG, and polyamidoamine dendrimer (dendrimer) for siRNA delivery targeting liver cancer therapy.^[^ ^162^ ^]^ The GA–PEG–GO–dendrimer (GPGD) of ≈100 nm obtained good stability, low toxicity, high transfection efficiency, and induced negligible hemolysis according to an in vitro test based on HepG2 cells. They observed significant cell uptake of GPGD–antivascular endothelial growth factor A (VEGFa) siRNA by HepG2 cells and decreased expression of VEGFa in both mRNA and protein levels, which indicated gene silencing (Figure 15c). In vivo tests on mice models demonstrated obvious growth inhibition of tumor tissues, and the MTT assay and histology analysis confirmed the safety of such a complex.

### 5.7 Immunotherapy

Immunotherapy revolutionized cancer treatments by transferring strategy from killing cancer cells directly to improving the natural immune defences. Leukocytes such as B and T cells^[^ ^11^ ^]^ and closely related natural killer (NK) cells^[^ ^206^ ^]^ are the main targets in immunotherapy. It has been noted that immune cell communication and activation were controlled by immunological synapses.^[^ ^206^ ^]^ The interactions of immunological synapses were based on clusters of cell surface molecules in size range of 10–400 nm. Previously, monoclonal antibodies like Rituximab^[^ ^206^ ^]^ or EB6^[^ ^207^ ^]^ were utilized to activate B cells or NK cells. However, the activation was weak as the antibodies are unbound soluble reagents. Therefore, developing antibody nanoclusters based on acellular soluble reagents would be more efficient, and graphene materials with suitable sizes would be better.

**Figure 16:**
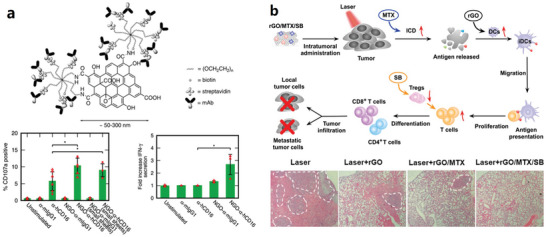
Immunotherapy is based on functionalized graphene materials. a) GO functionalization for activating NK cells and the average fold increase in the level of cell‐surface CD 107a and IFN‐*γ* secretion when treated with free mAb and mAb attached to GO. Reproduced with permission.^[^ ^206^ ^]^ Copyright 2018, American Chemical Society. b) Schematic representation of the synergistic effect of PTT and immune therapy induced by rGO‐MTX‐SB upon NIR laser and histology analysis of excised lungs ten days after different treatments, where the dotted circle areas indicate metastases. Reproduced with permission.^[^ ^11^ ^]^ Copyright 2020, Elsevier Ltd.

To activate NK cells, Loftus et al. utilized GO–PEG–biotin–streptavidin as a template to produce soluble antibody nanoclusters for NK cells via CD 16 receptors.^[^ ^206^ ^]^ Biotin groups were linked to the —NH_2_ of GO–PEG, and then streptavidin was coated on the complex. *α*‐hCD16 as antibodies for CD 16 receptors on NK were finally attached to GO–PEG–biotin–streptavidin. According to their results, by controlling the size of nanoclusters to 150 nm, more than 50% of primary NK cells bonded with such complex and further induced cellular activation (**Figure** **16**a). Degranulation of cytolytic granules (increased cell‐surface CD 107a) and IFN‐*γ* secretion assisted NK cell activation. In another synergistic method, T lymphocytes were activated in immunotherapy.

Zhou et al. reported a synergistic graphene nanosystem that combined PTT, drug delivery, and immunotherapy for cancer therapy and constructed an anticancer microenvironment to prevent recurrence.^[^ ^11^ ^]^ With rGO as a base plane, they loaded mitoxantrone (MTX) for chemical therapy, and SB‐431542 (SB) was attached as a TGF‐*β* inhibitor. With intravenous administration of such complex in mice bearing 4T1 tumor, the local primary tumor was destroyed upon NIR irradiation, and metastatic cancer was inhibited (Figure 16b). After treatment, more than 70% of mice survived, and a specific immunity was obtained to resist rechallenged tumors. Evidence from increased infiltration of CD8^+^ T lymphocytes also confirmed the immunological functions.

## 6 Conclusions, Challenges, and Outlook

Functionalized graphene materials have the potential to create breakthroughs in various fields of biomedical application. This review first introduces and evaluates six typical graphene synthesis methods. It is demonstrated that bare graphene materials synthesized with the methods are unsuitable for direct application in biomedical fields, while functionalization provides better efficacy. Then, characterization techniques, including structural detection and functional analysis, are summarized. The structural information, including morphology, thickness, lateral size, functional groups, and functional properties such as dynamic behavior, biodistribution, thermal stability, and various unique properties, have been described. Subsequently, basic functionalization methods to modify the properties of bare graphene and further functionalization methods that provide more functionalities to graphene materials are discussed based on the classification of covalent and noncovalent interactions, followed by the evaluation of toxicity and biocompatibility of the as‐produced graphene materials. The interaction mechanism between graphene and cells, five routes inducing cytotoxicity in vitro, the biodistribution of different graphene materials in vivo, histological changes induced by inflammatory or immune responses, and mitigation of toxicity are discussed. The hemolytic potential, platelet activation, platelet aggregation, and plasma coagulation are further discussed. On the other hand, the mechanism of toxicity and biocompatibility, biomedical applications, including imaging techniques, drug delivery, biosensor, tissue engineering, photothermal therapy, gene delivery, and immunotherapy based on various functionalized graphene materials, are introduced.

While the graphene industry has been developing rapidly in recent years, several issues and challenges remain to be addressed for better technological progress. First, the repeatability and consistency of graphene properties, such as size, thickness, and the number of functional groups, vary despite similar synthesis and functionalization methods. These variations would influence cell uptake and induce differences in cell viability. Therefore, aiming at biomedical applications, maintaining repeatability, and reducing variance are challenges that must be overcome. Reina et al. also proposed a lack of standard production criteria in lateral size, aggregation states, and oxidation states of graphene materials for biomedical applications.^[^ ^208^ ^]^ Based on this, it is suggested that material names, structural and functional characterization results, synthesis methods, functionalization methods, and recommended processing methods should be provided by the supplier or completed by researchers to achieve better horizontal comparison and standardization in the industry. If targeting the biomedical application of graphene materials, except for the above information, biocompatibility, toxicity, and biodegradation information are needed.

Second, many research groups obtained excellent therapeutic results based on mice treated with graphene materials in vivo. However, less attention was given to the control of treatment dose, nanoparticle concentrations, and influence of extrinsic stimulus to conform to preclinical safety pharmacology guidelines of regulatory agencies such as the FDA, ICH, and the EMA.^[^ ^42^ ^]^ Thus, maintaining the therapeutic effects while following these standards during the research and development of graphene materials as biomedicine would also be a challenge. Researchers are recommended to consider quality control in the synthesis process, safety evaluation in animal experiments, efficiency analysis in clinical research, and regulations for multidisciplinary. The criterions applied in products that FDA/ICH/EMA approved for commercial use could be a good guidance for graphene‐based products. For instance, for graphene‐based MRI contrast agents, evaluation from physiochemical properties, toxicity, biocompatibility, biodegradation, and in vitro phantom MRI based on the requirement for FDA approval instead of only imaging quality would be more comprehensive than most of the research in MRI contrast agent. It needs to be mentioned that different criterion is applied when facing specific application directions, and though GMP regulations in different regions have different principles and contents, comprehensive evaluation of graphene material cytotoxicity, sensitization, irritation, acute toxicity, material pyrogenicity, subchronic toxicity, genotoxicity, implantation, hemocompatibility, chronic toxicity, carcinogenicity, nephrotoxicity, and degradation are recommended if aiming at commercial biomedical applications.^[^ ^166^ ^]^

Third, the exact mechanism of why similar graphene materials cause different effects on different cell lines is unclear, hindering further study of graphene materials for molecular medicines. Although assumptions are proposed, the lack of verification based on suitable detection methods is challenging. Successful biomedical applications based on graphene materials, especially the long‐term and stable application, still require a combination of theoretical mechanisms (molecular dynamics simulation, first‐principles calculation, and metabonomics/proteomics/genetic omics study, etc.) and experimental phenomenon (high‐resolution real‐time imaging, cell viability assay, dynamic LS/FCM analysis, etc.). Future work should focus on studies of the interaction between graphene materials and different cell lines. In conclusion, the challenges need to be addressed soon.

The current scope of graphene materials in biomedical applications has been covered in many topics but still expanding. Several prospects are proposed for future work. Graphene materials have been developed and examined to function as contrast agents in enhancing imaging quality,^[^ ^25^ ^]^ improving imaging speed,^[^ ^6^ ^]^ and reducing harm to the human body.^[^ ^102a^ ^]^ Since graphene materials possess a large surface area, which may support multifunctionalization, a multimode imaging contrast agent could be developed based on graphene. For drug delivery, synergistic effects are more efficient than the simple delivery of drugs; thus, combining drug delivery with PTT immunotherapy would offer a better therapy. In biosensing and gene delivery for diagnosis, in vitro diagnosis dominates the market share. There is still not much research on in vivo diagnosis that achieves real‐time and dynamic evaluation, as the dissipation of fluorescence signals in deep tissue blocks the development of in vivo detection. Perhaps measurements based on spectrum shifts of mid‐NIR can be a potential solution.^[^ ^174^ ^]^ Applying medical cosmetology based on graphene materials functionalized with hyaluronic acid would be a potential research area for tissue engineering. Moreover, PTT would generally require furless conditions in the region of interest (ROI); thus, patients need to remove their hair if they have a brain tumor. Also, the bone penetration of laser would be another problem when treating brain tumors. Minimally invasive PAI applying fibers with micrometer‐diameter can combine biomedical imaging and PTT based on preadministrated graphene and suitable laser. Regarding the theoretical mechanism study of graphene interactions with cells, a deep learning/machine learning potential field that achieves highly accurate calculation comparable to ab initio molecular dynamics simulation and as fast as the potential empirical field is recommended.^[^ ^209^ ^]^ Though the training in the deep learning/machine learning potential field would be time‐consuming, an accurate study of the mechanism involves graphene materials entering into various cell membranes, graphene materials interaction with nucleotide, the surface energy of graphene materials with different sizes/thickness/functional groups, etc. would be beneficial for design experiments.

To summarize, research in graphene materials for biomedical applications is still in its infancy but promising. They illustrate great potential if current challenges can be tackled and their unique properties can be properly and readily utilized.

## Conflict of Interest

The authors declare no conflict of interest.
